# Solvent-induced structural and optical transformations in a hybrid copper phosphate framework

**DOI:** 10.1039/d6ce00295a

**Published:** 2026-05-25

**Authors:** Isis P. Carmona-Sepúlveda, Adelaide R. Kerenick, Julie L. Fenton

**Affiliations:** a Department of Chemistry, The Pennsylvania State University University Park PA 16802 USA fenton@psu.edu

## Abstract

Crystalline materials that undergo coupled structural and optical transformations in response to external stimuli are of broad interest in solid-state chemistry. Here, we report a crystalline hybrid copper phosphate, Cu_4_(4,4′-bipy)_4_(H_2_PO_4_)_4_·6H_2_O, that exhibits solvent-programmable structural and optical switching driven by dehydration and methanol uptake. The material crystallizes with undercoordinated Cu(i) centers in a porous framework stabilized by π–π stacking and hydrogen bonding. Upon heating, dehydration induces a reversible color change from yellow to deep red, accompanied by a crystallographic transformation from a low-symmetry triclinic phase to a higher-symmetry monoclinic phase with distorted tetrahedral Cu coordination. Variable temperature X-ray diffraction and UV-vis spectroscopy correlate this transformation with a red-shift in the metal-to-ligand charge transfer (MLCT) band. Exposing the dehydrated phase to dry methanol drives a different symmetry-breaking crystallographic transformation, in which ordered, directional methanol-framework interactions stabilize trigonal planar Cu(i) centers and produce a distinct cyan color. Together, these results demonstrate how solvent identity, hydrogen bonding directionality, and coordination geometry can be crystallographically coupled to achieve multiple optical responses and switching in a single hybrid phosphate material. This work establishes hybrid phosphate frameworks as a platform for post-synthetic structural and optical switching, accessing crystalline phases that are unreachable through traditional synthetic routes.

## Abstract 2

Hydration, dehydration, and methanol uptake switch a hybrid Cu(i) phosphate framework between three crystalline phases and three colors, linking solvent-driven structural change directly to optical response.

## Graphical abstract

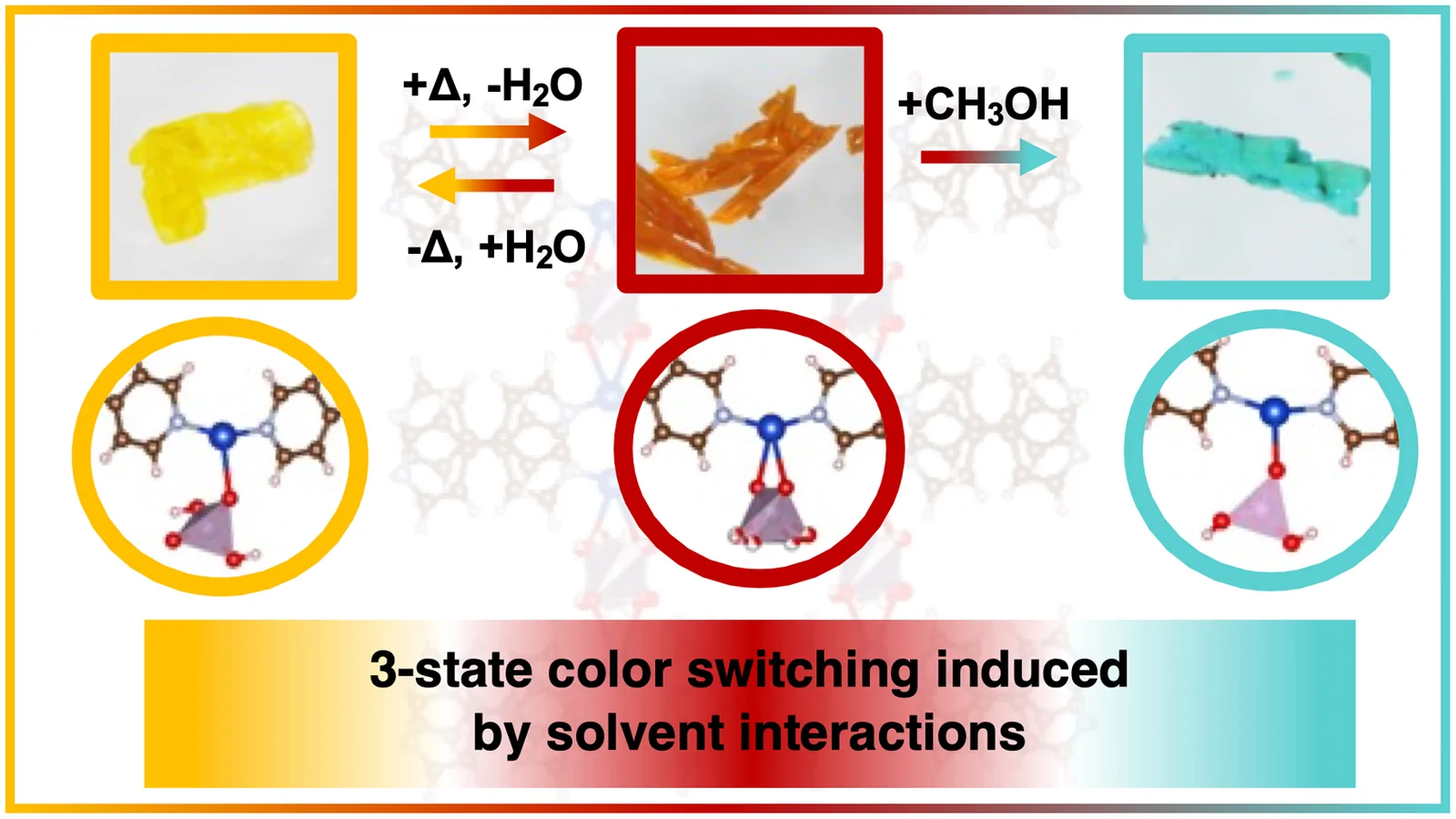

## Introduction

Materials that respond predictably to external stimuli are of broad interest in solid-state and materials chemistry.^1–4^ Thermochromic materials, which exhibit temperature-dependent color changes, exemplify this behavior and are widely studied as model systems for understanding structure–property relationships in solids.^1,5^ Such behavior occurs across compositionally diverse systems, including polymers, liquid crystals, hybrid nanocomposites, and inorganic oxides.^6–9^ The mechanisms driving thermally-induced color changes vary widely with structure and composition.^10,11^ Polymer systems typically rely on reversible chemical transformations like protonation/deprotonation or isomerization.^7,12^ For example, spiropyran units undergo reversible ring-opening upon heating, producing distinct color changes used in temperature-sensitive coatings and sensor technologies.^13^ In contrast, inorganic solids frequently exhibit phase transitions that alter metal coordination environments.^14,15^ VO_2_ is a classic example, switching from an insulating monoclinic phase to a metallic rutile-type structure, which has been exploited in smart window applications.^9,16^

Hybrid materials merge the tunability of molecular components with the stability of inorganic frameworks, yielding unique emergent properties that can transcend purely organic or inorganic solids. Optically responsive hybrids with diverse architectures, including coordination polymers, layered hybrids, halide perovskites, and metal–organic frameworks (MOFs), have been reported.^12,14,17–19^ Hybrid materials exhibit thermochromic mechanisms that combine features of both organic and inorganic systems, coupling structural and chemical transformations to temperature-driven color change. For example, layered hybrid lead halide perovskites exhibit dynamic color changes across a large continuum of colors, enabled by reversible phase transitions and hydrogen-bond mediated ion shuttling.^20,21^ The modular architectures and well-defined crystallinity of MOFs allow color changes to be directly correlated with structural changes; for instance, Zr-based UiO-66 changes from white to yellow upon dehydration, and Co-based frameworks transition from purple to pink during spin-state changes.^22,23^ Copper-based hybrids are particularly intriguing because Cu centers offer accessible redox states and flexible coordination geometries, enabling temperature-driven changes in various materials.^6,24^ For instance, copper carboxylate frameworks exhibit color changes upon heating or dehydration, shifting from greenish-blue in the hydrated state to deep blue when water molecules are removed.^25,26^ This transition is driven by the loss of coordinated water and subtle distortions in the Cu–O bonding environment, which modify ligand-to-metal charge transfer pathways and produce a visible thermochromic response. Layered phenylmethylammonium (PMA) copper halides ((PMA)_2_CuX_4_, X = Br or Cl) change from green at room temperature to yellow at elevated temperature, related to thermal lattice expansion and increased electron–phonon coupling.^27^ In many such systems, guest molecules or solvent play a critical role in stabilizing specific coordination environments or framework symmetries, providing a pathway to tune optical responses through subtle structural reorganization. Collectively, these systems highlight the versatility of hybrid architectures for designing responsive materials with tunable optical properties.

Metal phosphates are a structurally diverse class of materials that have long been studied for their stability, ion exchange capabilities, and catalytic properties.^28–30^ Inorganic phosphates are well known for their structural diversity, chemical robustness, and capacity for ion exchange and guest inclusion.^31,32^ Building on this foundation, hybrid metal phosphate systems introduce organic ligands into the coordination sphere of the metal center, effectively disrupting the continuity of the all-inorganic framework and enabling new structural motifs and functional behaviors. This substitution transforms the rigid, fully inorganic lattice into a more modular architecture, which combines the stability of phosphate networks with the chemical versatility of molecular ligands. Like their inorganic analogs, the resulting architectures are often porous, containing hydrophilic channels with numerous sites for dynamic hydration, hydrogen bonding, or ion exchange.^33–36^ Varying the metal identity, its coordination geometry, and the nature of the ligand allows for fine-tuning the framework connectivity and its resultant properties. This has recently been observed in other transition metal coordination polymers and MOF systems, where solvent inclusion and exclusion processes drive access to new structural states and emergent properties not observed in the as-synthesized phases.^37–39^ By leveraging the robustness of phosphate frameworks and the functionality of metal–organic interactions, hybrid metal phosphates offer a complementary platform to other crystalline hybrid solids like hybrid metal halides and MOFs for designing multifunctional materials, for designing multifunctional, stimuli-responsive materials. Despite these attributes, solvent-driven structural and optical responsiveness in hybrid metal phosphate frameworks remain comparatively underexplored, particularly in systems that preserve crystallinity and framework integrity through the transformation.

Here we report the synthesis and characterization of a crystalline hybrid copper phosphate, Cu_4_(4,4′-bipy)_4_(H_2_PO_4_)_4_·6H_2_O, that undergoes solvent-driven crystallographic and optical transformations. The framework contains undercoordinated Cu(i) centers linked by 4,4-bipyridine (bipy) and phosphate ligands into a porous three-dimensional lattice containing structure-stabilizing water molecules. Controlled dehydration induces a reversible thermochromic response, while subsequent methanol uptake enables access to alternative symmetry, coordination states, and optical response. Together, these transformations demonstrate how solvent identity, hydrogen-bonding interactions, and coordination changes can be leveraged to modulate structure and optical response within a single hybrid phosphate framework.

## Experimental

Complete experimental details are available in the SI.

### Synthesis of Cu_4_(4,4′-bipy)_4_(H_2_PO_4_)_4_·6H_2_O

In a 40 mL septum-capped vial, Cu(NO_3_)_2_·3H_2_O (0.5 mmol), and 4,4′-dipyridyl (0.25 mmol) were dissolved in a solvent mixture of dimethylformamide (DMF, 4 mL) and distilled H_2_O (12 mL). After this, H_3_PO_4_ 85% (1.6 mL) was slowly added into the solution and stirred until a clear blue solution was obtained, approximately 5 minutes after addition. The solution was heated to 140 °C for 24 h until yellow plate-like crystals were observed. The vial was then removed from the heat and slow cooled ambiently. The crystals were collected by vacuum filtration, washed with distilled water and ethanol to remove residual solvent, and air dried at room temperature. Samples were stored in vials for further characterization. Yield: 90%.

### Dehydration and methanol-absorption

The as-synthesized crystals were loaded into a round-bottom flask and desolvated under dynamic vacuum at 80 °C for 15 minutes, until a color change was observed from yellow to red. The flask was then removed from heat and allowed to cool to room temperature under N_2_. Dry MeOH was subsequently injected into the flask, and the crystals were allowed to soak for 10 minutes, during which a complete color change to yellow was observed. The resulting material was transferred to a vial and stored under ambient conditions for further characterization.

### Variable temperature single-crystal X-ray diffraction

Data collections were performed using the Rigaku Oxford Diffraction Synergy Custom diffractometer comprising of Rigaku MicroMax 007 rotating anode Cu Kα radiation X-ray generator (*λ* = 1.5418 Å) operating at 40 kV 30 mA and HyPix-Arc 150 photon counting detector. A suitable single crystal was selected and mounted on a quartz fiber with cyanoacrylate glue. Data collection was performed at 173–400 K temperature ranges using the Cobra Oxford Cryosystem. Data reduction was performed with the CrysAlisPro software using both numerical absorption and Gaussian correction methods. Using Olex2, the structure was solved with the SHELXT structure solution program using intrinsic phasing and refined with the SHELXL refinement package using least-squares minimization methods.

### Variable temperature powder X-ray diffraction

Data was collected on a PANalytical Empyrean® theta–theta X-ray diffractometer equipped with an Anton Parr HTK1200 non-ambient chamber and a copper (Cu) line source [Kα1–2 = 1.540598/1.544426 Å] X-ray tube at 45 kV and 40 mA on a 240 mm radius with a step size of 0.0167° from 5–60° 2-theta. The sample was finely ground into a powder and mounted into an alumina crucible (16 mm diameter and 0.8 mm deep). The incident optics consisted of a Bragg–Brentano HD® Cu optic fitted with 0.04 rad. Soller slits, a 4 mm beam mask, 1/8° and 1/2° divergence, and anti-scatter slit respectively. The diffracted optics included a X'Celerator® detector with a 2.1223° active length in scanning line mode with a 1/2° programmable anti-scatter slit and 0.04 rad Soller slits. Experimental data was collected as the sample was heated from room temperature to 135 °C, with 60 °C min^−1^ temperature increments, and 10 min hold times before each scan collection.

### Variable temperature diffuse reflectance UV-vis spectroscopy

Data was collected using a PerkinElmer Lambda 950 equipped with the 150 nm integrating sphere detector. The sample compartment was equipped with the Praying Mantis (Harrick) diffuse reflection accessory, and the sample was introduced in the temperature chamber mounted with CaF_2_ windows. The sample was diluted with BaSO_4_ and finely ground into a powder using a mortar and pestle prior to loading it inside the cell. The temperature was controlled using the automatic temperature controller (ATC, Harrick) autotuned at 200 °C. Pure BaSO_4_ was used for the baseline. Data collection was performed under a flow of dry synthetic air (25 mL min^−1^) between 30 °C and 210 °C with a heating rate of 5 °C min^−1^, collecting a spectrum every 10 °C steps with 5 min hold at the desired temperature before data acquisition. Data was collected between 200–1000 nm with 2 nm intervals, and 0.32 s integration with a 4 nm slit. The lamp changeover was set at 319.2 nm, while monochromator and detector changes was set at 860.8 nm.

## Results and discussion

### Synthesis and structural characterization

**Fig. 1:**
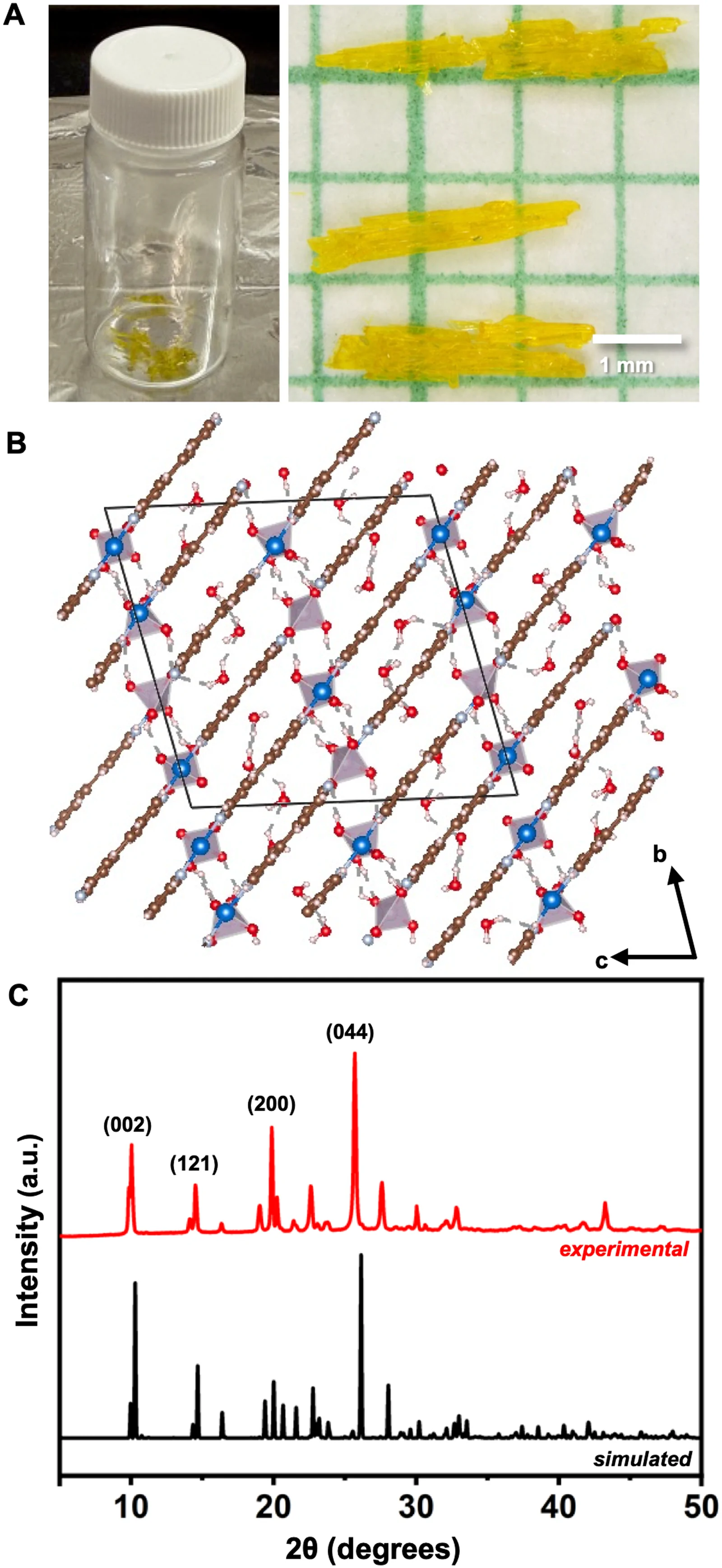
Characterization of the as-synthesized single crystals of Cu_4_(4,4′-bipy)_4_(H_2_PO_4_)_4_·6H_2_O, including (A) photograph and optical microscope image; (B) rendering of the structure solution obtained from single-crystal X-ray diffraction (XRD), and (C) indexed experimental powder XRD pattern compared to a simulated structure from the structure solution. The simulated pattern in (C) was obtained from a structure solution without a solvent mask, which better accounts for the electron density of disordered water in the pores.

**Fig. 2:**
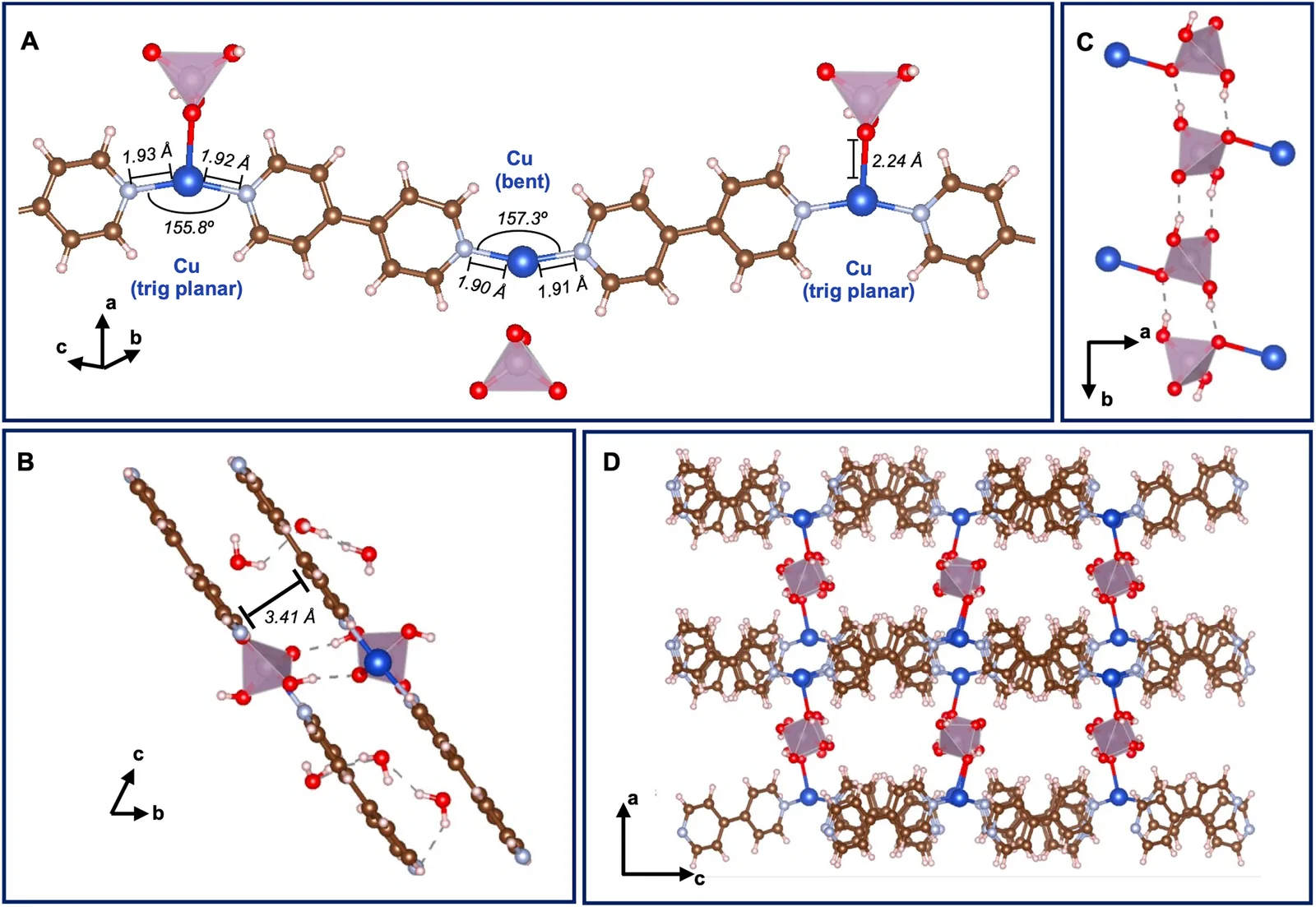
Details of the structure solution obtained from single-crystal X-ray diffraction for Cu_4_(4,4′-bipy)_4_(H_2_PO_4_)_4_·6H_2_O. The structure of the Cu-bipy coordination polymer is shown in (A), highlighting the two distinct trigonal planar and bent Cu coordination sites. In (B), a view highlighting π–π stacking between neighboring coordination polymer chains. Panel (C) highlights the hydrogen-bound chains of phosphate anions that stabilize the *b*-axis of the structure. Viewing the structure along the *b*-axis in (D) reveals the structural pore size and shape. Disordered water molecules occupy the empty space in the rectangular pores and have been omitted for clarity from (D).

Single crystals of Cu_4_(4,4′-bipy)_4_(H_2_PO_4_)_4_·6H_2_O were synthesized by dissolving copper(ii) nitrate and 4,4′-bipyridine (bipy) in a mixture of DMF and H_2_O at room temperature, followed by addition of H_3_PO_4_. The solution was heated to 140 °C for 24 hours and ambiently cooled, forming yellow, plate-like crystals that were isolated by filtration (Fig. 1A and S1). Single crystal X-ray diffraction (scXRD) was used to obtain structural information from the as-synthesized crystals. The unit cell is shown in Fig. 1B, and detailed crystallographic information is available in Table S1. The structure crystallizes in the triclinic *P*1̄ space group, with unit cell dimensions of *a* = 8.8724(1) Å, *b* = 16.9583(3) Å, *c* = 17.6998(4) Å. Key structural features are highlighted in Fig. 2. The asymmetric unit is composed of four distinct copper sites, with two observed geometries. Three of the copper sites are coordinated to two nitrogen atoms from separate bipy ligands and a pendant oxygen from an [H_2_PO_4_]^−^ anion, forming a distorted trigonal planar building unit [Cu(4,4′-bipy)_2_(H_2_PO_4_)]_*n*_ (Fig. 2A). On the fourth copper site, two 4,4′-bipyridines are bonded to the copper center in a slightly bent geometry, forming a second [Cu(4,4′-bipy)_2_]_*n*_ structural unit (Fig. 2A). This copper site is not directly bound to any phosphate groups. The site appears to weakly interact with phosphate oxygens, with extended Cu⋯O distances of 2.617 Å and 2.722 Å, considerably longer than reported Cu–O bond lengths.^41,42^

Low-coordinate Cu(i) environments, while less common than the more typical tetrahedral geometries, are established in systems where steric or electronic factors limit the coordination number. Linear, two-coordinate Cu(i) centers have been structurally verified in complexes stabilized by sterically demanding N-heterocyclic carbene ligands, demonstrating that such low coordination numbers are synthetically accessible.^43,44^ Three-coordinate Cu(i) sites are likewise precedented, particularly in systems supported by bulky or strongly donating ligands that enforce trigonal geometries and disfavor higher coordination.^45,46^ Undercoordinated Cu(i) sites observed in porous or framework materials may also be stabilized by weak interactions with adjacent anions or solvent molecules, offering additional precedent for the low-coordinate Cu(i) environments present in this structure.^40,47^ In this system, the undercoordinated sites may similarly be stabilized by weak interactions with proximal phosphate anions and pore-occupying water molecules.

The two types of building units condense into a three-dimensional porous framework, with chemically distinct interactions stabilizing each dimension. In one dimension, metal–organic coordination bonds link alternating Cu and bipy moieties into planar 1D coordination polymers (Fig. 2A and B). The threads are arranged to promote face-on π–π interactions between bipy molecules on adjacent chains, forming extended supramolecular columns in a second dimension. The interchain distance (3.40 Å) is close enough to allow for effective π–π overlap, indicating that non-covalent supramolecular interactions play a key role in stabilizing the framework in this direction (Fig. 2B).^48^ The third dimension is defined by interactions among phosphate groups: copper-coordinated phosphate anions assemble into extended chains, formed by six-membered hydrogen-bonded rings of protonated –OH groups (Fig. 2C). Together, the elongated phosphate chains and stacked coordination polymers form porous channels, visible along the crystallographic *b*-axis (Fig. 2D). The approximately rectangular pores are framed on two sides by phosphate chains and on the other two by the edges of bipy ligands. The material displays a small measurable surface area as determined by nitrogen adsorption. The pore size distribution from these measurements reveals a pore size distribution centered around 10 Å, which is qualitatively aligned with the expected value of approximately 9 Å derived from the crystal structure (Fig. S2). Water molecules occupy the structural pores and appear to engage in hydrogen bonding with nearby phosphate groups. However, their precise positions could not be refined crystallographically due to mobility-related disorder, and the single-crystal structure, illustrated in Fig. 1 and 2, was accordingly refined using a solvent mask to account for the unresolved electron density in the pores, in accordance with standard practice for other porous framework solids.^49^ Although the water molecules do not directly coordinate to the metal centers, their presence influences local Cu coordination geometry through hydrogen-bonding interactions with proximal phosphate groups, acting in concert with the π–π stacking interactions that stabilize the framework. Pore solvent plays a subtler templating role, one that becomes apparent upon its removal, as discussed in the following section.

Powder X-ray diffraction (pXRD) confirms the phase-purity of the bulk material and the presence of disordered water in the structure. An experimental pattern collected from finely ground single crystals was compared to a simulated pattern derived from the single crystal structure solution as detailed above (Fig. S3). The peak positions in both patterns are in reasonable agreement, though the experimental data, collected at room temperature, shows slight shifts to lower angles consistent with the effect of thermal expansion. More notably, deviations in relative peak intensities are observed, particularly for the (100), (102), and (200) reflections at 10.0°, 14.3°, and 20.0° 2*θ*, respectively. We hypothesized that these differences may be due to the contribution of disordered solvent molecules in the experimental samples, the effect of which would be exacerbated for lower angle reflections.^50,51^ To evaluate this, the experimental pXRD pattern was compared to a simulated pattern from a structure solution refined without a solvent mask (Fig. 1C), which retains the electron density from the disordered water molecules in the pores. Since the unit cell dimensions are consistent between the solvent-masked and unmasked models, the peak positions in both simulated patterns show similar agreement with the experimental data. However, the unmasked model shows improved alignment in relative peak intensities with the experimental pattern. This comparison supports the presence of water molecules in the as-synthesized material and highlights their influence on the diffraction pattern, despite not being part of the extended framework.

X-ray photoelectron spectroscopy (XPS) suggests that copper atoms in the compound are exclusively monovalent (Cu^+^), consistent with a charge-neutral stoichiometry determined by single-crystal XRD. XPS measurements were performed on a polycrystalline sample of the as-synthesized compound. The survey spectrum (Fig. S4) displays characteristic signals for Cu, P, O, N, and C, consistent with the expected elemental composition. The spectral features observed for regions corresponding to N 1s, C 1s, O 1s, and P 2p are consistent with the expected presence of phosphate and 4,4′-bipyridine moieties in the compound. To confirm the oxidation state of copper, high-resolution scans were acquired in regions corresponding to the Cu 2p_3/2_ and Cu LMM transitions, observed at 931.8 eV and 915.5 eV, respectively. The oxidation state can be determined for Cu by calculating the Auger parameter, defined as the sum of the binding energy of Cu 2p_3/2_ and the kinetic energy of the Cu LMM Auger electron. The measured Auger parameter of 1847.3 eV closely matches the reported value for Cu_2_O (1849 eV), confirming the presence of Cu^+^ in the sample. The formation of Cu(i) from a Cu(ii) precursor under these conditions is consistent with well-precedented *in situ* reduction during solvothermal synthesis in DMF. Under hydrothermal conditions and in the presence of acid, DMF undergoes partial hydrolysis to generate formate, which can act as an *in situ* reductant. This mechanism has been confirmed spectroscopically in analogous solvothermal syntheses of Cu(i)-containing materials from Cu(ii) salts.^52,53^

UV-vis spectroscopy further supports the assignment of Cu^+^ and clarifies the origin of the material's yellow color. The diffuse-reflectance UV-vis spectrum (Fig. S5) lacks the d–d transition expected near 750 nm for Cu^2+^, consistent with the filled d^10^ configuration of Cu(i), which precludes ligand-field transitions. Instead, the optical response is dominated by charge-transfer processes. A high-energy feature centered near 300 nm arises from the π–π* transitions intrinsic of the bipyridine ligands, while a broad absorption band extending from 350 to 530 nm is characteristic of a metal-to-ligand charge transfer (MLCT) transition. The onset wavelength of this feature is approximately 526 nm (2.38 eV). The MLCT involves electron transfer from filled Cu(i) orbitals to the π* orbitals of bipyridine, producing the observed yellow color. The observed spectral features align with expectations for Cu(i) complexes paired with electron-rich bipyridine and phosphate ligands.^27,41,47^ The energy of the MLCT onset is therefore sensitive to the Cu(i) coordination environment: changes in coordination geometry, ligand binding mode, or the identity of donor atoms directly modulate the relative energies of the Cu d–donor and bipyridyl π*–acceptor orbitals, providing a structural basis for the distinct optical responses observed across phases.

### Dehydration-induced structural reorganization and optical switching

Taken together, the as-synthesized structure contains a framework stabilized by a combination of metal–organic coordination bonds, π–π stacking, and phosphate-directed hydrogen bonding, with significant disorder introduced by water molecules which occupy the structural pores. While these water molecules do not appear to participate directly in metal coordination, their presence influences packing and symmetry. Removal of pore solvent therefore provides a direct means to probe whether these interactions merely occupy free volume or actively determine the preferred structural arrangement and copper coordination environments. To evaluate this, we examined the effect of controlled dehydration on the synthesized structure.

**Fig. 3:**
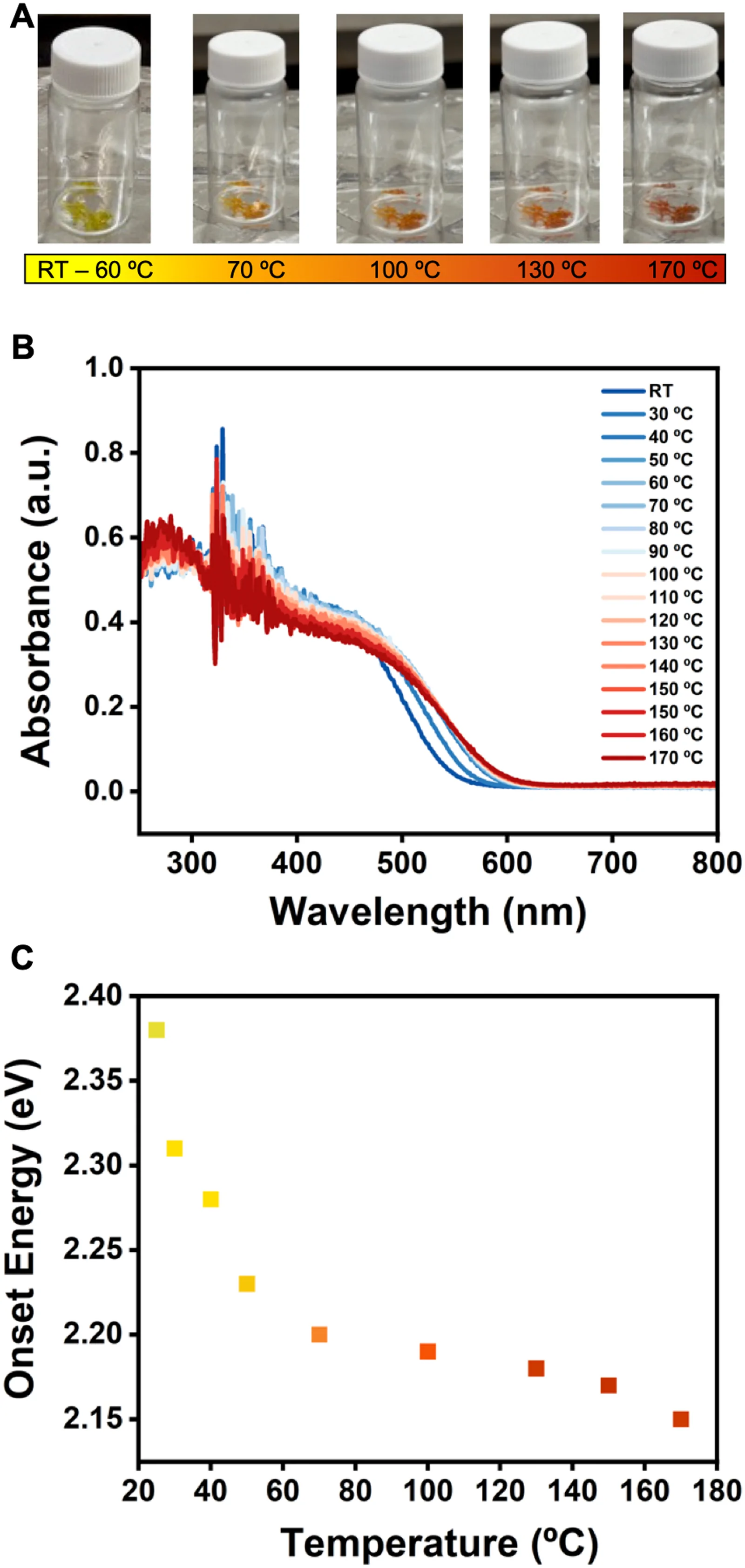
(A) Photographs depicting the material color changes, from yellow to deep red. The labeled temperatures indicate the readout from an external hot plate. (B) Diffuse-reflectance UV-vis absorption spectra collected at varied temperatures. (C) Absorption onset energy as a function of temperature.

The prepared compound displays reversible, temperature-dependent color changes that are directly coupled to framework dehydration. Upon heating from room temperature to moderate temperatures, the material undergoes a visible transition from yellow to deep red, which is fully reversible upon cooling in air (Fig. 3A, Videos S1 and S2). Heating above 170 °C results in irreversible color change, leaving the compound permanently dark green upon cooling. The color change behavior was monitored using variable temperature UV-vis absorption spectroscopy (Fig. 3B). Across the temperature range where reversible color change occurs, the spectral shape remains relatively consistent, with no additional features observed up to 170 °C. The most notable change in the spectra between room temperature and 170 °C is a prominent red shift in absorption onset as temperature increases, which is aligned with the observed yellow to red color change. The absence of the Cu^2+^ d–d transition near 750 nm verifies retention of the 3d^10^ state. Beyond 170 °C, the spectrum shows an intensified feature below 300 nm and a new band near 750 nm (Fig. S6), indicating copper oxidation at high temperature. The onset energy of absorption (Fig. 3C), determined by extrapolating the linear portion of the main absorption feature, reveals three regimes of color change: a rapid redshift in onset energy between room temperature and 70 °C, a plateau from 70 °C to ∼130 °C, and above 140 °C, a gradual redshift in values again. The steep initial slope suggests that initial solvent-related changes within the framework dominate the reversible color response, whereas more extensive chemical changes at higher temperatures, such as copper oxidation, account for the irreversible behavior. The accompanying red-shift in the MLCT onset with increasing temperature likely reflects changes in the local environment of Cu(i) associated with dehydration.

The compound is thermally stable up to 170 °C and decomposes through a multi-step pathway, with the loss of water coinciding with the onset of the observed color change. Thermogravimetric analysis (TGA) was performed to understand the thermal stability of the as-synthesized single crystals (Fig. S7). Between 25 °C and 100 °C, a 7.5% mass loss is observed, consistent with the water content occupying the structural pores (Table S2). The mass loss plateaus off between 100 °C and 170 °C, when a second mass loss event begins. Between 170 °C and 400 °C, approximately 45% of the material is lost, corresponding to the complete volatilization of the bipy ligands. This temperature range is consistent with the reported thermal stability of bipy in similar framework materials.^24,54^ Above 400 °C, the mass loss plateaus, leaving a final residual mass of approximately 48%, which is aligned with the total mass attributed to phosphate groups and copper in the original structure. The final product of the decomposition process is presumed to be an oxidized, dehydrated Cu(ii) phosphate species.^55^ Taken together, the reversible color changes observed visually and by UV-vis spectroscopy occur between room temperature and 170 °C, and based on TGA data, begins to occur with the loss of structural water. Beyond 170 °C, the compound decomposes into oxidized products, leading to a loss of the framework and its reversible color change behavior.

**Fig. 4:**
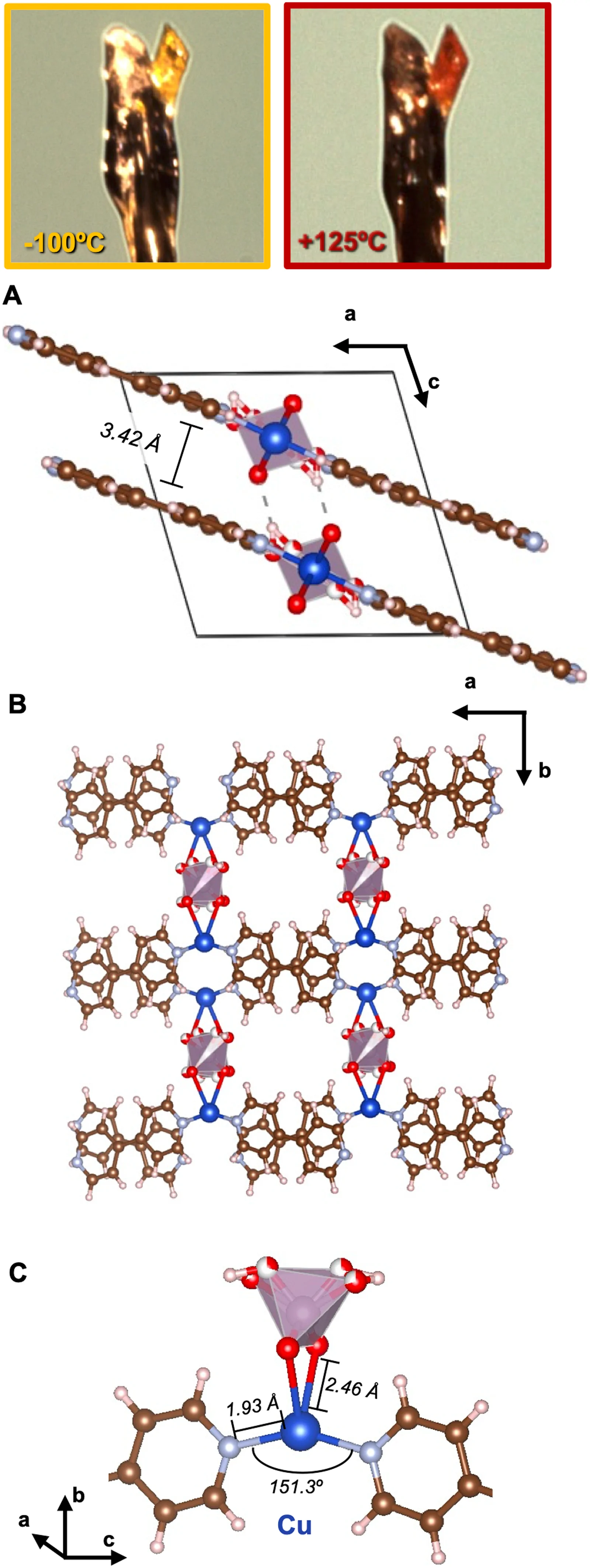
Results from variable temperature single-crystal X-ray diffraction experiment. Photos at top show color change of crystal during experiment, yellow under cryogenic conditions at −100 °C and red at elevated temperatures (+125 °C) upon dehydration. Panel (A) contains the unit cell of Cu(4,4′-bipy)(PO_3_), highlighting the distance between neighboring coordination polymers. (B) Viewing the structure along the *c*-axis highlights the pseudo-rectangular pores present in the structure. Panel (C) shows the single distorted tetrahedral coordination site, bond distances, and angles for Cu in the dehydrated structure.

To directly correlate dehydration with framework reorganization and copper coordination changes, variable-temperature single crystal X-ray diffraction (scXRD) analysis was employed to probe the structural evolution of a single crystal across the dehydration regime. A hydrated crystal was mounted on a glass fiber with heat-resistant glue. An initial data set at −100 °C confirmed the hydrated structure as described above. The temperature was then raised gradually to 125 °C, collecting data sets at intermediate temperature. Refinement at intermediate temperatures yielded structures with poor statistics, with difficulties attributed to the combination of disordered water and increased thermal lattice vibrations. In contrast, the final data set, collected at 125 °C where water is presumed absent from the structure, resulted in a structure solution with reasonable refinement statistics. This structure closely resembles the low-temperature structure: coordination polymers of alternating copper atoms and bipy ligands extend in one dimension, π–π stacking defines a second, and hydrogen bound phosphate chains stabilize the third, forming pseudo-rectangular pores. As expected, no electron density is observed in the pores, indicating complete water removal and resulting in improved data quality and refinement statistics. Water loss is accompanied by increased structural symmetry, with the original low-symmetry *P*1̄ trigonal space group transformed into a higher symmetry monoclinic *P*2/*c* space group and a correspondingly smaller unit cell (*a* = 8.5755(3) Å, *b* = 8.7283(2) Å, and *c* = 8.5690(3) Å). Beyond water removal, the most notable structural change is in the copper coordination environment: the high-temperature structure contains a single crystallographic site, consistent with the increase in symmetry (Fig. 4). In this site, Cu adopts a distorted tetrahedral geometry, coordinated by two bipy ligands and two oxygens from phosphate anions. This change in coordination geometry, from the mixed trigonal planar and undercoordinated environments of the hydrated phase to a single distorted tetrahedral site with direct Cu–O(phosphate) bonding, modifies the Cu d-orbital energies and their overlap with the bipyridyl π* acceptor levels, consistent with the observed red-shift in MLCT onset and the yellow-to-red color change. The structural transformation was further corroborated by variable temperature powder XRD measurements. These data (Fig. S8), shown alongside simulated patterns for the hydrated and dehydrated structures, reveal a clear progression between the two phases. A systematic shift to lower diffraction angles is observed as temperature increases, consistent with the transition from the lower-symmetry triclinic structure to the higher-symmetry monoclinic phase. Overall, the temperature-dependent evolution of the powder patterns mirrors the single-crystal results and confirms that removal of solvent from the pores drives reorganization into the higher-symmetry phase.

Dehydration is accompanied by a symmetry-increasing single-crystal-to-single-crystal transformation rather than a framework collapse, indicating that the solvent-free framework represents a favorable structural arrangement of the host lattice.^56,57^ In this high-symmetry phase, the framework converges to a single crystallographic Cu site, with Cu adopting a distorted tetrahedral coordination environment. The emergence of a unique, well-defined dehydrated structure places this phase as a potential structural nexus from which alternative, lower-symmetry arrangements may be accessed through solvent inclusion. This further supports our hypothesis that the included solvent molecules stabilize the framework through intermolecular interaction, where disordered hydrogen-bonding interactions template a lower-symmetry hydrated triclinic phase. The loss of these interactions upon solvent removal promotes a dynamic structural rearrangement to a less distorted Cu coordination environment and results in a higher-symmetry monoclinic lattice, where π–π stacking interactions predominate.

Motivated by this observation, we investigated whether re-introduction of guest molecules could direct the dehydrated framework into distinct structural states by modifying symmetry, copper coordination geometry, and optical response. Dehydrated crystals were exposed to a range of common off-the-shelf solvents varying in size, polarity, and hydrogen-bonding ability, including methanol, ethanol, butanol, and isopropyl alcohol. In all cases using off-the-shelf (*i.e.*, water-containing) solvents, the crystals rapidly reverted to a yellow color, consistent with rapid and kinetically favored water uptake and regeneration of the hydrated phase. In contrast, when submerged in rigorously dried, air-free conditions, the dehydrated crystals remained red indefinitely in all solvents examined except methanol.

**Fig. 5:**
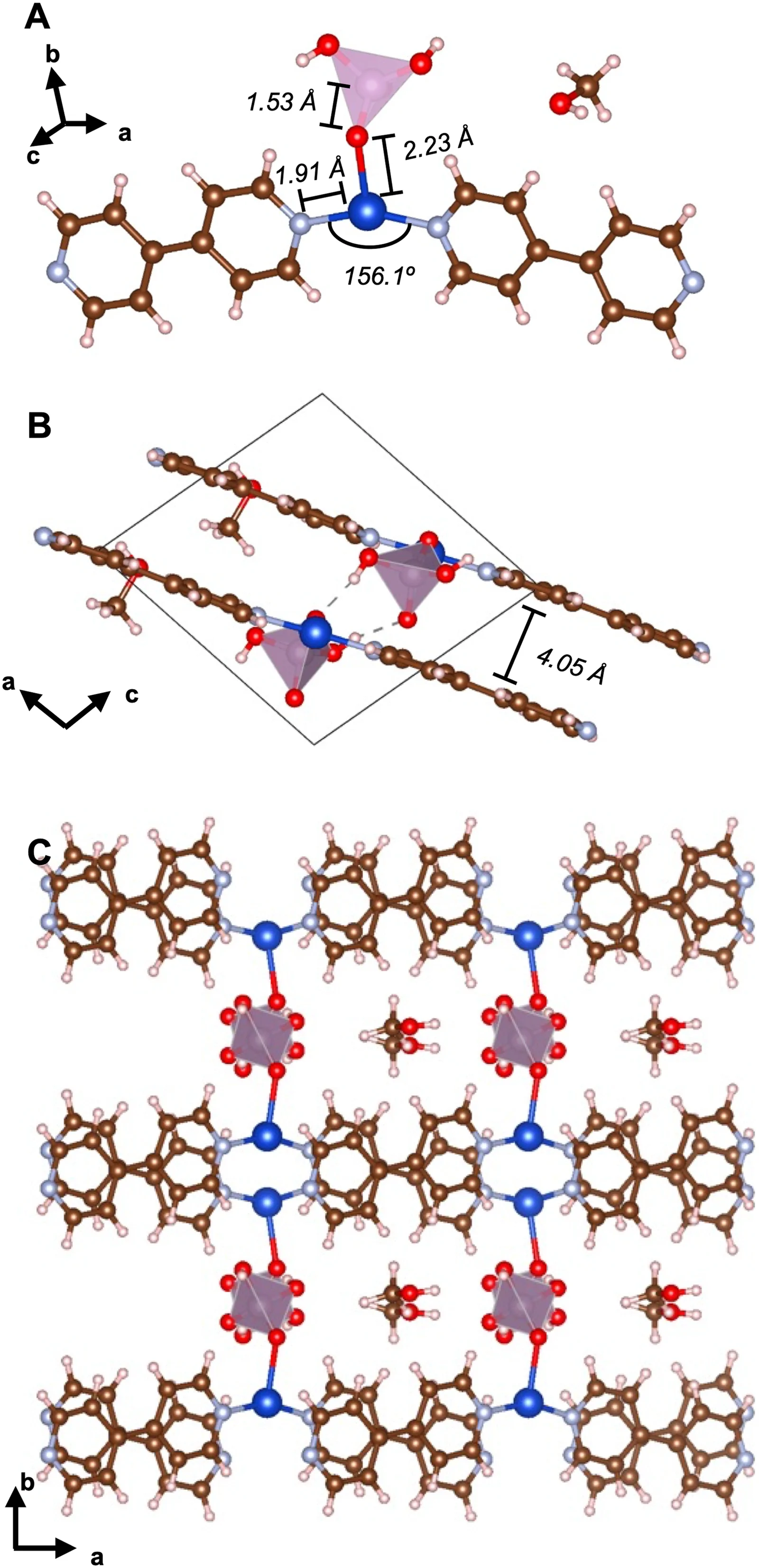
Structure solution obtained from the single-crystal X-ray diffraction of the methanol-absorbed crystals: Cu(4,4′-bipy)_2_(H_2_PO_4_)(MeOH). The asymmetric unit is shown in (A), highlighting the Cu trigonal planar coordination with two bipys and one phosphate anion. In (B), phosphate–phosphate interactions and π–π stacking are also observed. Viewing the structure along the *c*-axis in (C) shows the structural pore that is occupied by methanol molecules that interact through hydrogen-bonding and van der Waals interactions.

Exposure to dry methanol induced a rapid color change from red to green-yellow within approximately 5 minutes, indicating stabilization of a new solvent-directed structural state. Single-crystal X-ray diffraction performed immediately after solvent uptake shows that the methanol-absorbed crystals adopt a non-centrosymmetric monoclinic *Pc* space group (Table S3). The asymmetric unit contains a single crystallographic Cu site coordinated by two bipy ligands and one oxygen from the pendant phosphate anion (Fig. 5). Methanol resides within the pores and engages in directional hydrogen bonding with terminal phosphate groups, resulting in a more ordered and anisotropic solvent-framework interaction than observed for water and stabilizing a distinct Cu(i) coordination environment. Consistent with this assignment, the optical response of the methanol-absorbed phase is dominated by ligand-centered π–π* and MLCT transitions, with no high-wavelength features associated with Cu(ii), confirming the retention of the Cu(i) oxidation state (Fig. S9).

**Fig. 6:**
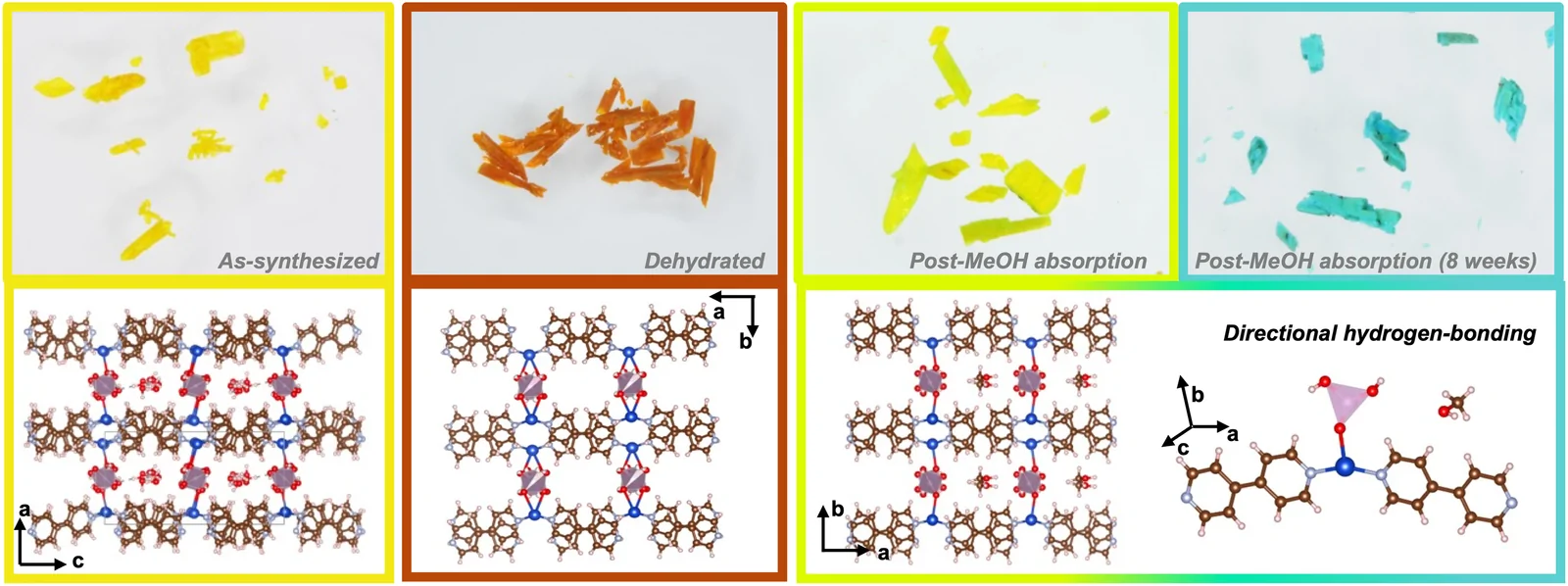
Optical micrographs of representative single crystals in the hydrated, dehydrated, and methanol-absorbed phases, illustrating the distinct color changes associated with each structural transformation driven by solvent identity.

Over time, the methanol-absorbed crystals undergo a gradual color change from green-yellow to cyan (Fig. 6). Initial hypotheses attributing this behavior to solvent mobility or framework reorganization were ruled out by scXRD performed on samples stored for two months under ambient conditions, which revealed no structural or Cu coordination changes (Table S3). Though the bulk framework structure remains unaffected over this period, XPS performed on these same stored samples (Fig. S10) reveals partial surface oxidation to Cu(ii), consistent with the known behavior of Cu(i) after prolonged air exposure. Unlike the hydrated phase, the methanol-absorbed crystals show no thermally-induced color switching on heating, suggesting an enhanced thermal stability attributable to stronger and more specific methanol-framework interactions, including directional hydrogen-bonding and van der Waals interactions between the methyl group and the pore scaffold.^58,59^ Consistent with this interpretation, TGA of the methanol-absorbed crystals (Fig. S11) reveals a gradual mass loss extending to 170 °C, rather than a sharp, low-temperature dehydration previously observed. The elevated onset temperature and broadened mass-loss profile indicate multiple interaction strengths and diffusion-limited solvent removal from the pores, confirming that methanol is stabilized within the framework.^58^

Methanol inclusion triggers a further solvent-induced transformation within the same single-crystal to single-crystal framework flexibility observed during dehydration, but with a different structural outcome (Table S19). Methanol uptake drives a symmetry-breaking transformation from the centrosymmetric dehydrated phase to a non-centrosymmetric structure. This reduction in symmetry is consistent with ordered and directional incorporation of methanol within the pores, in contrast to the more disordered interactions of water. The progression of space groups across the series (*P*1̄ → *P*2/*c* → *Pc*), identifies the dehydrated phase as a crystallographic branching point, from which solvent identity dictates access to two distinct lower-symmetry arrangements with different properties.

## Conclusion

We report a hybrid copper phosphate framework, Cu_4_(4,4′-bipy)_4_(H_2_PO_4_)_4_·6H_2_O, that undergoes solvent-driven structural transformations coupled to distinct optical responses. The as-synthesized material features undercoordinated Cu(i) centers bonded to 4,4′-bipyridine and phosphate ligands, forming a porous 3D architecture stabilized by π–π stacking and hydrogen bonding. Controlled dehydration induces a reversible yellow-to-red thermochromic response, which temperature-dependent X-ray diffraction and UV-vis spectroscopy correlate to a transition in copper coordination geometry that modulates the metal-to-ligand charge transfer band. Removal of pore solvent triggers a symmetry-increasing single-crystal-to-single-crystal transformation into a high-symmetry framework with distorted tetrahedral Cu(i) centers. In contrast, methanol inclusion drives a symmetry-breaking transformation to a non-centrosymmetric phase, accompanied by a progressive color change to cyan and stabilization of trigonal planar Cu(i) centers. Together, the three-stage evolution of the hydrated, dehydrated, and methanol-absorbed phases illustrates the central role of solvent identity in directing framework symmetry, local coordination geometry, and optical response. Hydration templates a low-symmetry structure with multiple Cu coordination environments, dehydration reveals a higher-symmetry phase with distorted tetrahedral Cu(i) sites, and methanol inclusion drives a symmetry-breaking transformation that stabilizes trigonal planar Cu(i) centers. These solvent-dependent coordination changes directly govern observed optical behavior and establish solvent identity as a key parameter in controlling crystallographic symmetry and electronic structure in hybrid phosphate frameworks.

## Author contributions

I. P. C.-S. and J. L. F. planned the experiments, analyzed the data, and wrote the manuscript, with input from A. R. K. I. P. C.-S. conducted synthesis and structural characterization of all samples. I. P. C.-S. and A. R. K. conducted methanol uptake experiments. A. R. K. conducted surface area analysis. J. L. F. supervised the project. All authors have given approval to the final version of the manuscript.

## Conflicts of interest

There are no conflicts of interest to declare.

## Supplementary Material

CE-028-D6CE00295A-s001

CE-028-D6CE00295A-s002

CE-028-D6CE00295A-s003

CE-028-D6CE00295A-s004

## Data Availability

The data supporting this article have been included as part of the supplementary information (SI), which contains full experimental and synthetic details, crystallographic summary information, and additional characterization data. Video files showing sample heating to 170 °C (Video S1) and cooling from 170 °C (Video S2) are also provided as separate files. Crystallographic data for this study have been deposited with the Cambridge Crystallographic Data Centre (CCDC) under deposition numbers 2496744, 2496747, and 2539939. Supplementary information is available. See DOI: https://doi.org/10.1039/d6ce00295a. CCDC 2496744, 2496747, and 2539939 contains the supplementary crystallographic data for this paper.^60*a*–*c*^
