# Supplementary material for: Solvent-induced structural and optical transformations in a hybrid copper phosphate framework
Source: CrystEngComm. 2026 May 25;28(24):3785–96. doi: 10.1039/d6ce00295a (PMC13220123; doi:10.1039/d6ce00295a)
Supplement: CE-028-D6CE00295A-s001 [file CE-028-D6CE00295A-s001.pdf]

Supplementary Information (SI) for CrystEngComm.
This journal is © The Royal Society of Chemistry 2026

Solvent-Induced Structural and Optical Transformations in a Hybrid Copper Phosphate Framework

Isis P. Carmona-Sepúlveda, Adelaide R. Kerenick, and Julie L. Fenton*

Department of Chemistry, The Pennsylvania State University, University Park, PA 16802, United States

Supplementary Information

Correspondence Address
Professor Julie L. Fenton<br>Department of Chemistry<br>Pennsylvania State University<br>University Park, PA 16802 (USA)<br>Email: fenton@psu.edu

Table of Contents

A. Materials and instrumentation S-2

B. Synthesis details S-5

C. Additional characterization data S-6

D. Additional crystallographic information S-17

E. References S-31

A. Materials and Instrumentation.

Materials

All reagents and solvents were commercially sourced as used without any further purification. $Cu(NO_3)_2•3H_2O$ (99+%), 4,4'-dipyridyl (98%), and o-phosphoric acid (85% w/w) were purchased from Sigma-Aldrich. *N,N*-dimethylformamide (DMF) (99+%) was purchased from Fisher Scientific. Solvents, including ethanol (EtOH) were of analytical grade. Dry MeOH was prepared by stirring over activated 3 Å molecular sieves under a continuous N_2 purge for 2 hours and stored under a N_2 atmosphere prior to use.

Instrumentation

Powder X-ray Diffraction. Powder X-ray diffraction (pXRD) patterns were obtained at room temperature on a Bruker D2 Phaser diffractometer using Cu Kα radiation (λ = 1.54056 Å). Scan rates were 0.25 s/step with a step size of 0.02º within a 5-60º range. PXRD scans were collected after single crystals were finely ground in a powder stored in vials at room temperature under normal atmosphere and loaded onto a zero-backgroun silicon plate.

Single Crystal X-ray Diffraction. Single crystal X-ray diffraction (scXRD) data collections were performed using the Rigaku Oxford Diffraction (ROD) Synergy Custom diffractometer comprising of Rigaku MicroMax 007 rotating anode Cu Kα radiation X-ray generator (λ = 1.5418 Å) operating at 40 kV 30 mA and HyPix-Arc 150 photon counting detector. A suitable single crystal was selected and mounted on a quartz fiber with cyanoacrylate glue. Data collection was performed at 173-400 K temperature ranges using the Cobra Oxford Cryosystem. Data reduction was performed with the CrysAlisPro software using both numerical absorption and Gaussian correction methods. Using OLEX2, the structure was solved with the SHELXT structure solution program using intrinsic phasing and refined with the SHELXL refinement package using least-squares minimization methods. Restraints on anisotropic displacement parameters were applied to ensure physically reasonable thermal ellipsoids for disordered atoms. Hydrogen atoms were expressed on organic species in the most probable geometric positions once the relevant carbon and nitrogen atoms were assigned and refined. The three-dimensional crystal structure was modeled using the Visualization for Electronic and Structural Analysis (VESTA) software.

Variable Temperature Diffuse Reflectance UV-Vis Spectroscopy. Data was collected using a PerkinElmer Lambda 950 equipped with the 150 nm integrating sphere detector. The sample compartment was equipped with the Praying Mantis (Harrick) diffuse reflection accessory and the sample was introduced in the temperature chamber mounted with CaF_2 windows. The sample was diluted with $BaSO_4$ and finely grinded into a powder using a mortar and pestle prior to loading it inside the cell. The temperature was controlled using the automatic temperature controller (ATC, Harrick) autotuned at 200 ºC. Pure $BaSO_4$ was used for the baseline. Data collection was performed under a flow of dry synthetic air (25 mL/min) between 30 ºC and 210 ºC with a heating rate of 5 ºC/min,

collecting a spectrum every 10 ºC steps with 5-min hold at the desired temperature before data acquisition. Data was collected between 200-1000 nm with 2 nm intervals, and 0.32 s integration with a 4 nm slit. The lamp changeover was set at 319.2 nm, while monochromator and detector changes was set at 860.8 nm. Data was converted from diffuse reflectance to pseudo-absorption data using the Kubelka-Munk function.

Variable Temperature Powder X-ray Diffraction. Powder X-ray diffraction patterns were collected at 45 kV and 40 mA on a 240mm radius Panalytical Empyrean® theta-theta X-ray diffractometer equipped with an Anton Parr HTK1200 non-ambient chamber and a copper (Cu) line source [Kα1-2 = 1.540598/1.544426 Å] X-ray tube. Data was collected with a step size of 0.0167° from 5-60° 2-theta. The sample was finely ground into a powder and mounted using a top-load method into an alumina crucible (16 mm diameter and 0.8 mm deep). The incident optics consisted of a Bragg-Brentano HD® Cu optic fitted with 0.04 rad. Soller slits, a 4 mm beam mask, 1/8° and 1/2° divergence, and anti-scatter slit respectively. The diffracted optics included a X'Celerator® detector with a 2.1223° active length in scanning line mode with a 1/2° programmable anti-scatter slit and 0.04 rad Soller slits. Experimental data was collected as the sample was heated from room temperature to 135 ºC, with 60 ºC/min temperature increments, and ten-minute hold times before each scan collection.

Scanning Electron Microscopy and Energy Dispersive X-ray Spectroscopy. Crystal morphology and elemental mapping were collected using a Thermoscientific Apreo S Scanning Electron Microscope with a tungsten filament and fitted with an Oxford instruments Ultim Max EDS detector. Crystals were selected and mounted on stainless steel studs with carbon tape. For the elemental analysis, at least three regions of at least three unique crystals were selected to confirm compositional homogeneity.

***Thermogravimetric Analysis*.** Thermogravimetric analysis (TGA) was carried out on a TA Instruments Discovery TGA 550. Samples were loaded in platinum pans, approximately weighing 2 mg. Measurements were collected under a flow of nitrogen (90 mL/min) from 25-500 ºC with a heating rate of 10 ºC/min and 5 min isotherms.

X-ray Photoelectron Spectroscopy. XPS experiments were performed using a Physical Electronics VersaProbe III instrument equipped with a monochromatic Al Kα x-ray source (hν = 1486.6 eV) and a concentric hemispherical analyzer. Charge neutralization was performed using both low energy electrons (<5 eV) and argon ions. The binding energy axis was calibrated using sputter cleaned Cu (Cu $2p_{3/2}$ = 932.62 eV, Cu $3p_{3/2}$ = 75.1 eV) and Au foils (Au $4f_{7/2}$ = 83.96 eV). Peaks were charge referenced to CH_x band in the carbon 1s spectra at 284.8 eV. Measurements were made at a takeoff angle of 45° with respect to the sample surface plane. This resulted in a typical sampling depth of 3-6 nm (95% of the signal originated from this depth or shallower). Quantification was done using instrumental relative sensitivity factors (RSFs) that account for the X-ray cross section and inelastic mean free path of the electrons. On homogeneous samples major elements (>5 atom%) tend to have standard deviations of <3% while minor elements can be significantly higher. The analysis size was ~200µm in diameter.

Optical Microscopy. High-resolution images were obtained using a Nikon SMZ18 optical microscope.

Surface area and pore size measurement. Nitrogen physisorption experiments were performed using a Micromeritics 3Flex Surface Characterization Analyzer. The sample was weighed into a 12 mm flat-bottom sample tube, fitted with a check seal cap and hanging filler rod, then evacuated under dynamic vacuum using a Micromeritics VacPrep 061 unit. The degassing procedure for isotherm collection involved heating the sample to 120 °C at a ramp rate of 1.5 degrees/min, then holding at 120 °C for 48 hours under a pressure of 0.1 Torr. Prior to analysis, the sealed samples were reweighed and immediately transferred to the 3Flex sample ports. The nitrogen adsorption-desorption isotherm was measured at -196 °C (77 K) with the saturation pressure recorded at each data point. After the isotherm was completed, the analysis and ambient free space was measured with helium. For data analysis, Brunauer Emmett-Teller (BET) surface areas were calculated from the linear region of the isotherm at 77 K within the pressure range P/P_0 of 0.05 – 0.1. Pore size measurements were calculated using the Horvath-Kawazoe method fitted for a slit pore geometry within the pressure range P/P_0 of 0.003 – 0.1.

Room Temperature Diffuse Reflectance UV-Vis Spectroscopy. The absorption spectrum for the methanol-absorbed sample was collected as diffuse reflectance from 220-900 nm and converted to pseudo-absorption data using the Kubelka-Munk function in the native Shimadzu software. Data was collected on a Shimadzu UV-2600i UV-Vis spectrophotometer with an ISR-2600 Plus integrating sphere attachment.

B. Synthesis Details

Synthesis of $Cu_4(4,4'\text{-bipy})_4(H_2PO_4)_4 \cdot 6H_2O$. In a 40 mL septa-capped vial, $Cu(NO_3)_2 \cdot 3H_2O$ (0.5 mmol), and 4,4'-dipyridyl (0.25 mmol) were dissolved in a solvent mixture of DMF (4 mL) and distilled H_2O (12 mL). After this, H_3PO_4 85% (1.6 mL) was slowly added into the solution and stirred for at least 5 minutes until a clear blue solution was obtained. The solution was heated to 140 ºC for 24 h until yellow plate-like crystals were observed (yield ~90%). The vial was then removed from the heat to allow to cool under ambient conditions. These crystals were collected, washed with distilled water and ethanol to remove residual solvent, and air dried at room temperature. Samples were stored in vials for further characterization. Additional stoichiometric ratios and solvent mixtures were investigated (e.g. 1:2, 1:3, no DMF, no H_2O), but no crystallization was observed under these conditions.

Post-synthetic solvent absorption. Approximately 15 mg of the as-synthesized crystals were loaded into a round-bottom flask and desolvated under dynamic vacuum at 80 ºC for 15 minutes, until a color change was observed from yellow to red. The flask was then removed from heat and allowed to cool to room temperature under N_2. Dry MeOH was subsequently injected into the flask, and the crystals were allowed to soak for 10 minutes, during which a complete color change to yellow was observed. The resulting material was transferred to a vial and stored under ambient conditions.

C. Additional structural characterization

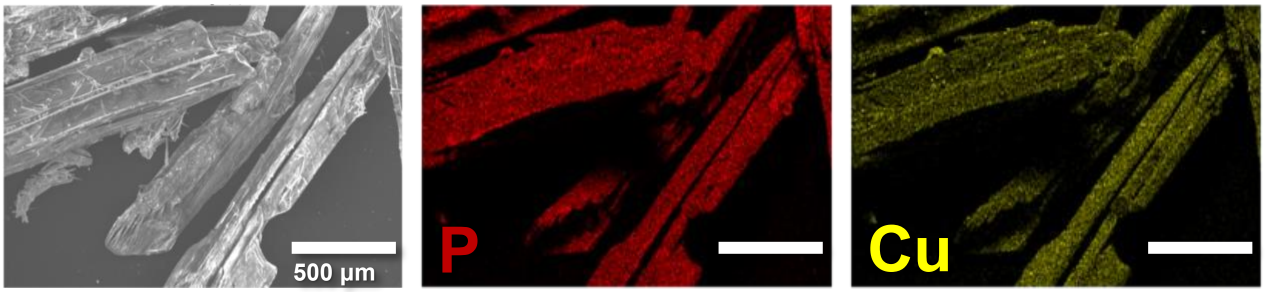

Figure S1. Scanning electron micrographs and elemental maps analyzing the composition of the as-synthesized $Cu_4(4,4'\text{-bipy})_4(H_2PO_4)_4\bullet 6H_2O$.

Table S1. Crystallographic details at different temperatures (hydrated and dehydrated).

**Temperature**	-100 ºC (173 K)	125 ºC (400 K)
**Empirical formula**	$C_{40}H_{37}Cu_4N_8O_{16}P_4$	$C_{10}H_{10}CuN_2O_4P$
**Wavelength**	Cu Kα (λ = 1.54184 Å)	
**Crystal system**	Triclinic	Monoclinic
**Space group**	$P\overline{1}$	$P2/c$
**Unit cell dimensions**	a = 8.87240(10) Å<br>b = 16.9583(3) Å<br>c = 17.6998(4) Å<br>α = 76.181(2)º<br>β = 89.867(2)º<br>γ = 89.996(2)º	a = 8.5755(3) Å<br>b = 8.7283(2) Å<br>c = 8.5690(3) Å<br>α = 90º<br>β = 105.924(4)º<br>γ = 90º
**Volume**	2586.04(8) $Å^3$	616.77(4) $Å^3$
**Z**	2	2
**Density (calc.)**	1.624 g/cm^3	1.705 g/cm^3
**Absorption coefficient**	3.656 mm^{-1}	3.832 mm^{-1}
**F(000)**	1276.0	320.0
**θ for data collection**	5.142º to 152.424º	10.134º to 151.916º
**Index ranges**	$-10 \leq h \leq 11$<br>$-18 \leq k \leq 21$<br>$-21 \leq l \leq 21$	$-9 \leq h \leq 10$<br>$-10 \leq k \leq 10$<br>$-10 \leq l \leq 8$
**Reflections collected**	34946	6161
**Independent reflections**	10260	1257
**Completeness**	99.9%	100%
**Data/restraints/parameters**	10260/18/655	1257/0/97
**GOOF**	1.068	0.999
**R_{int}**	0.0566	0.0342
**R indices [I>2σI)]**	0.1294	0.1053
**R indices (all data)**	0.1360	0.1088

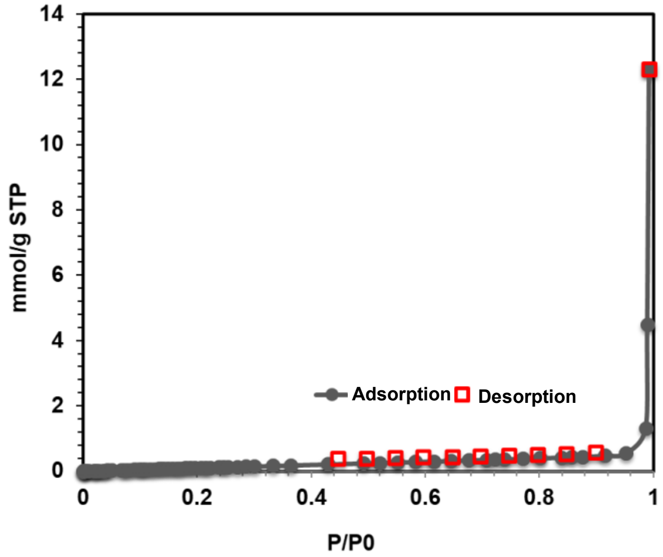

BET Report		Horvath-Kawazoe Report	
BET surface area:	16.5 ± 0.1 m²/g	Median pore width	12.291 Å
Slope:	4.44 ± 0.04 g/mmol	Maximum pore volume	0.003342 cm³/g
Y-intercept:	1.481 ± 0.007 g/mmol	at Relative Pressure	0.179186713
C:	3.996	Relative pressure range	1e-09 to 0.18
Qm:	0.16902 mmol/g		

Figure S2. Nitrogen absorption isotherm for dried sample of Cu_4(4,4'-bipy)$_4$(H_2PO_4)$_4$•6H_2O. Gray trace is adsorption; open red squares are desorption. The summary data computed from this isotherm, including BET surface area and median pore size, are given in the accompanying table.

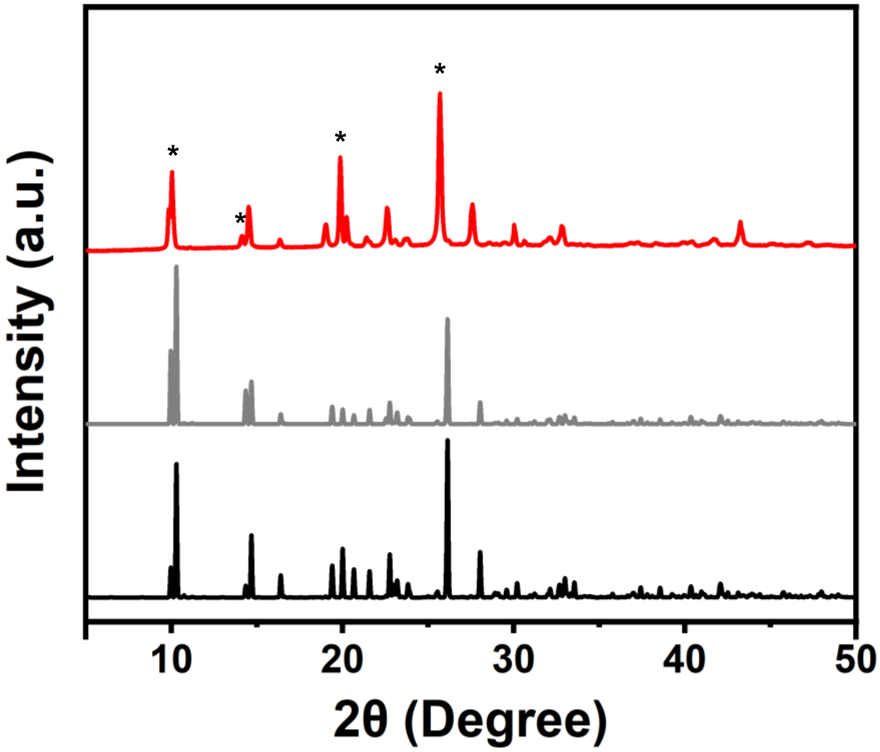

Figure S3. Powder X-ray diffractograms of simulated pattern without (black) and with (gray) solvent mask compared to the experimental pattern.

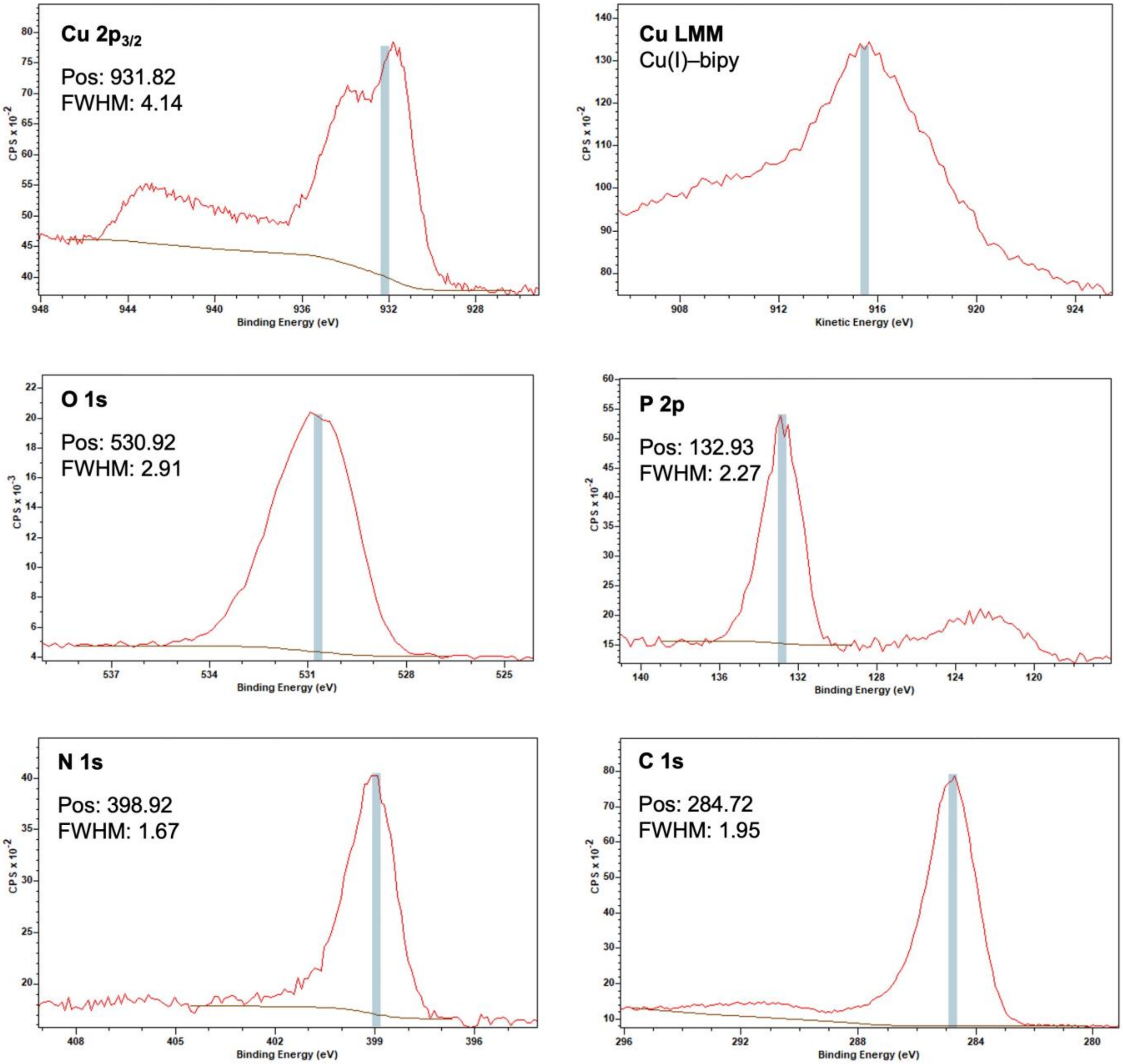

Figure S4. XPS binding energy spectra for $Cu_4(4,4'\text{-bipy})_4(H_2PO_4)_4{\bullet}6H_2O$ with the characteristic signals for Cu, P, O, N, and C.

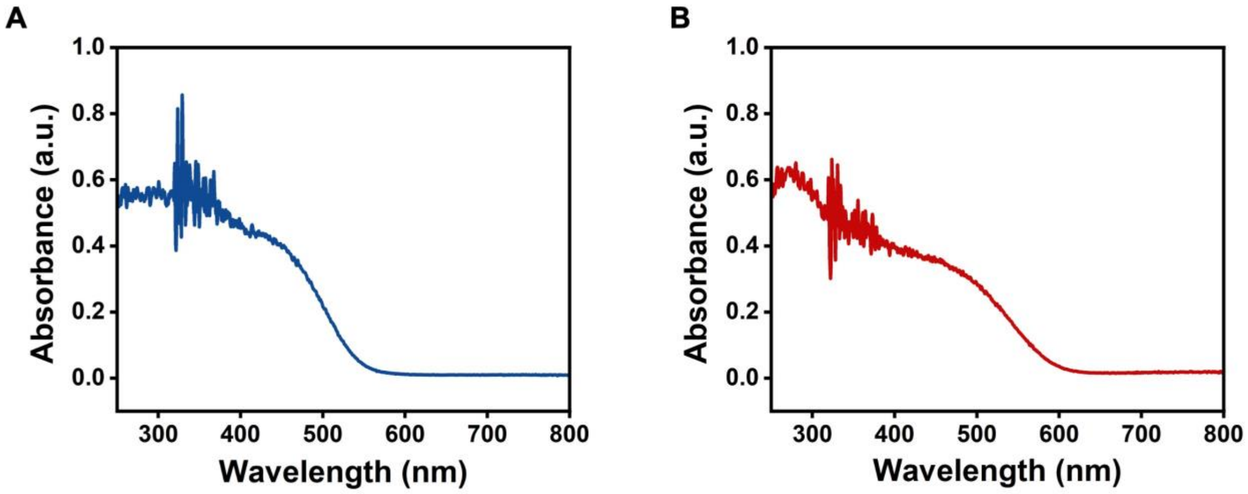

Figure S5. Absorption spectras of $Cu_4(4,4'\text{-bipy})_4(H_2PO_4)_4 \cdot 6H_2O$ at A) room temperature and B) 170 ºC.

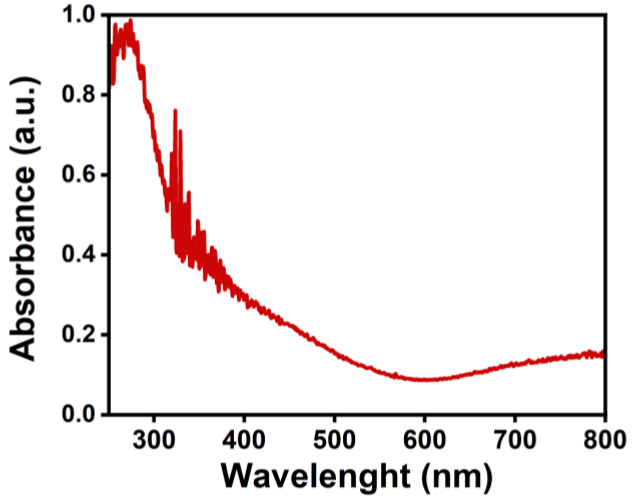

Figure S6. Absorption spectra of $Cu_4(4,4'\text{-bipy})_4(H_2PO_4)_4 \cdot 6H_2O$ at 250 ºC.

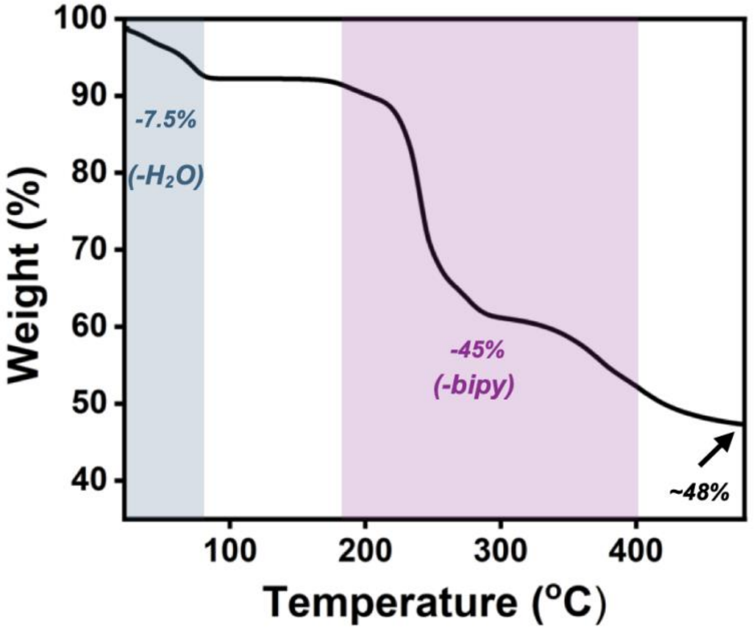

Figure S7. Thermogravimetric analysis (TGA) plot of as-synthesized single crystals. Regions corresponding to the loss of structural water and 4,4'-bipyridine ligands are highlighted in blue and purple, respectively. The final residual mass (approx. 48%) corresponds to residual oxidized copper(II) phosphate species as the final decomposition product. Experiment was performed under nitrogen.

Table S2. Theoretical and actual weight loss percentages with TGA for the as-synthesized single crystals of $Cu_4(4,4'\text{-bipy})_4(H_2PO_4)_4 \cdot 6H_2O$ (MW = 1,374.68 g/mol).

	Molecular weight (g/mol)	Total in compound	% of compound mass
4,4'-bipyridine	158.18 (4 mol)	624.72	45.4
H_2O	18.02 (6 mol)	108.12	7.9
Cu	63.55 (4 mol)	254.20	18.5
H_2PO_4	96.91 (4 mol)	387.64	28.2

Temperature (°C)	Experimental mass loss (TGA, %)	Hypothesized process
0-100	7.5	Loss of water from structural pores (theoretical: 7.9% of mass)
170-400	45	Volatilization of 4,4'-bipy ligands (theoretical: 45.4% of mass)
>400	minimal	remnant Cu(II) phosphate species (theoretical: 47.5% of mass)

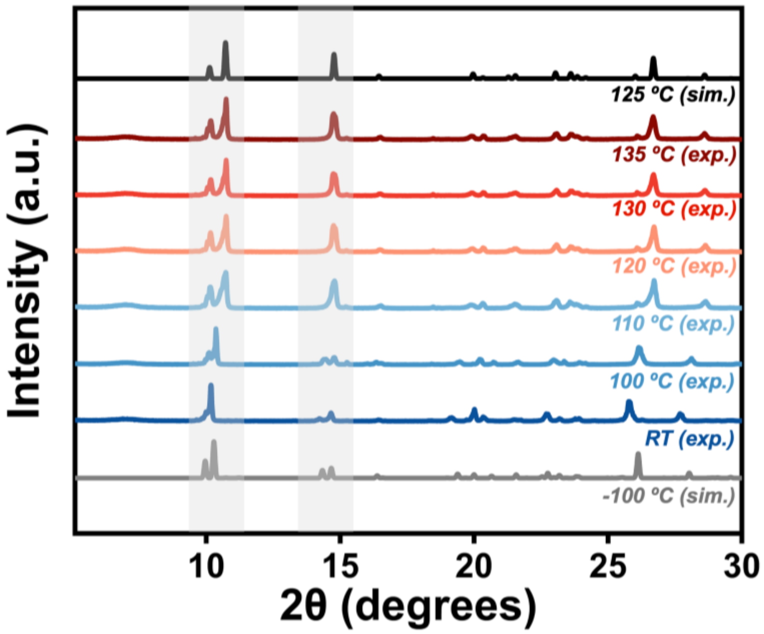

Figure S8. Experimental variable-temperature powder X-ray diffractograms, and simulated patterns at low and high temperatures. The highlighted peaks illustrate temperature-dependent structural transformations and symmetry changes.

Table S3. Crystallographic data of MeOH-absorbed single crystals as-intercalated and stored in ambient conditions for two months.

	Freshly intercalated	***Stored for two months***
**Temperature**	-100 ºC (173 K)	
**Empirical formula**	$C_{11}H_{14}CuN_2O_5P$	
**Wavelength**	Cu Kα (λ = 1.54184 Å)	
**Crystal system**	Monoclinic	
**Space group**	*P*c	
**Unit cell dimensions**	a = 8.9384(6) Å<br>b = 8.8927(5) Å<br>c = 8.4655(5) Å<br>α = 90º<br>β = 103.346(7)º<br>γ = 90º	a = 8.9149(9) Å<br>b = 8.8954(8) Å<br>c = 8.4547(8) Å<br>α = 90º<br>β = 103.373(10)º<br>γ = 90º
**Volume**	654.72(7) $Å^3$	652.29(11) $Å^3$
**Z**	2	2
**Density (calc.)**	1.769 g/cm^3	1.776 g/cm^3
**Absorption coefficient**	3.731 mm^{-1}	3.745 mm^{-1}
**F(000)**	356.0	
**θ for data collection**	9.946º to 149.784º	9.944º to 152.314º
**Index ranges**	$-11 \le h \le 10$<br>$-11 \le k \le 10$<br>$-6 \le l \le 10$	$-9 \le h \le 11$<br>$-10 \le k \le 10$<br>$-10 \le l \le 7$
**Reflections collected**	3318	6130
**Independent reflections**	1746	2066
**Completeness**	100%	
**Data/restraints/parameters**	1746/14/186	2066/6/209
**GOOF**	1.098	1.078
**R_{int}**	0.0415	0.0483
**R indices [I>2σI)]**	0.0510	0.0418
**R indices (all data)**	0.0556	0.0802
**Flack parameter**	0.32(8)	0.34(11)

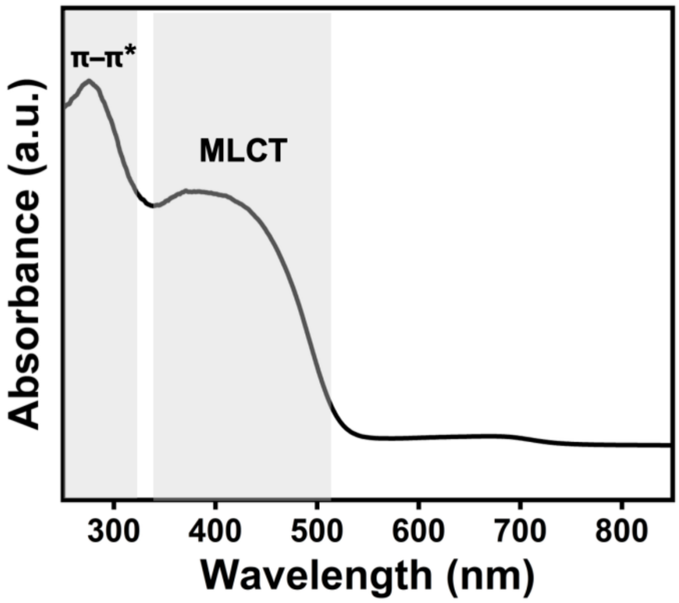

Figure S9. Absorption spectra of methanol-absorbed crystals at room temperature.

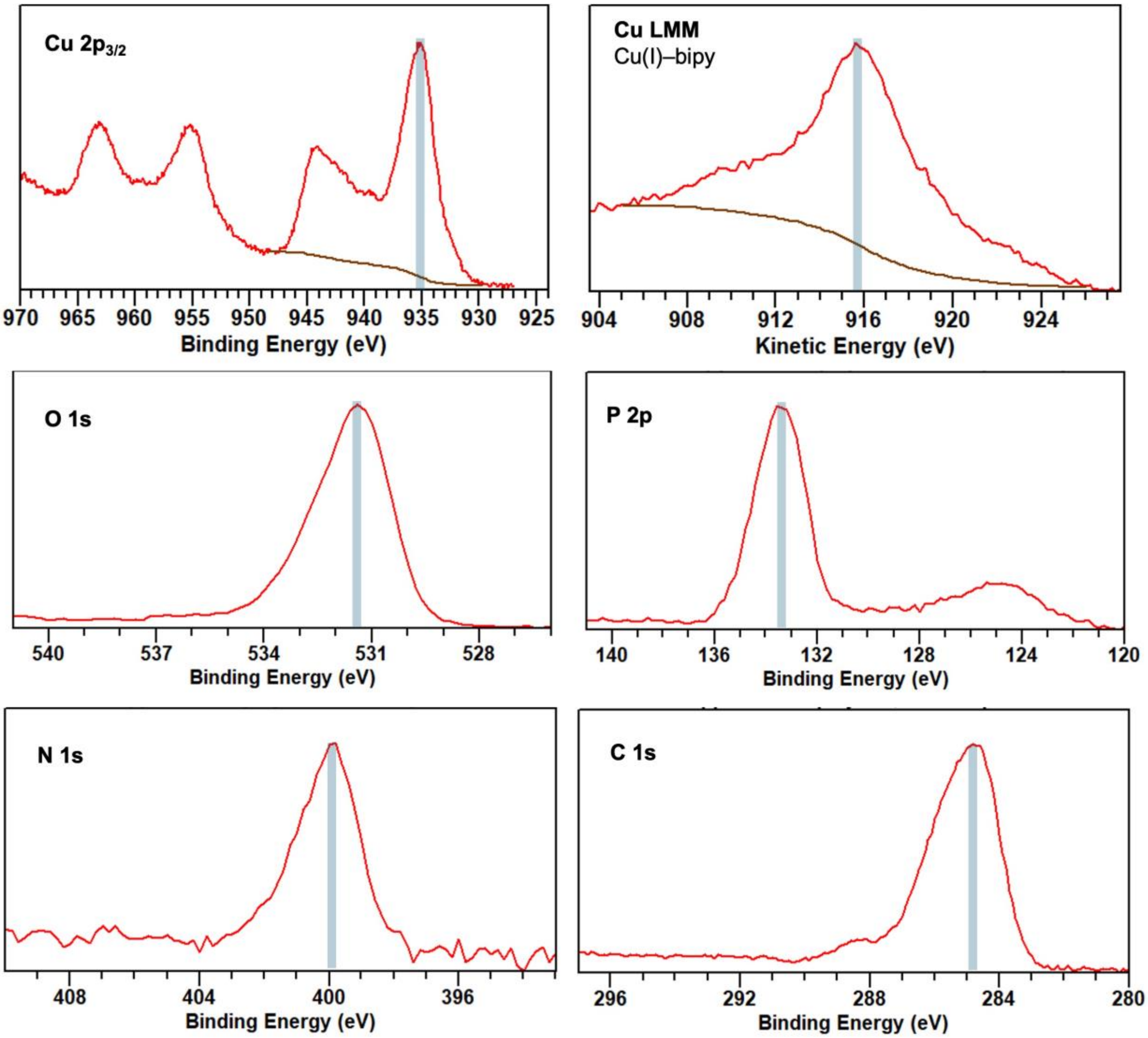

Figure S10. XPS binding energy spectra for the MeOH-absorbed crystals with the characteristic signals for Cu, P, O, N, and C after two months of storage. The Cu signal indicates the presence of both Cu(I) and Cu(II), suggesting partial surface oxidation after storage in ambient environments.

Table S4. Summary of crystallographic parameters, Cu coordination geometry, and optical absorption maxima in all three structural states.

Structural state	Hydrated	Dehydrated	MeOH-absorbed
**Unit cell dimensions**	a = 8.87240(10) Å<br>b = 16.9583(3) Å<br>c = 17.6998(4) Å<br>α = 76.181(2)º<br>β = 89.867(2)º<br>γ = 89.996(2)º	a = 8.5755(3) Å<br>b = 8.7283(2) Å<br>c = 8.5690(3) Å<br>α = 90º<br>β = 105.924(4)º<br>γ = 90º	a = 8.9384(6) Å<br>b = 8.8927(5) Å<br>c = 8.4655(5) Å<br>α = 90º<br>β = 103.346(7)º<br>γ = 90º
**Crystal system**	Triclinic	Monoclinic	Monoclinic
**Space group**	$P\bar{1}$	$P2/c$	Pc
**Cell volume**	2586.04(8) Å^3	616.77(4) Å^3	654.72(7) Å^3
**Cu coordination geometry**	Trigonal planar	Distorted tetrahedral	
**Optical absorption maxima**	330 nm	250 nm	260 nm

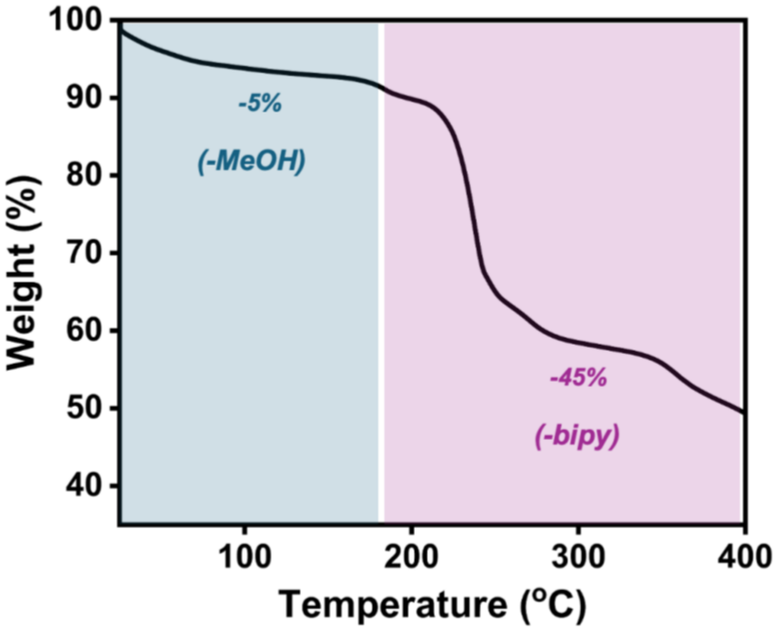

Figure S11. TGA plot of methanol-absorbed single crystals. Regions corresponding to the loss of structural methanol and 4,4'-bipyridine ligands are highlighted in blue and purple, respectively.

Table S5. Actual weight loss percentages of the methanol-absorbed crystals: Cu(4,4'-bipy)(H_2PO_4)(MeOH). (MW= 348.75 g/mol)

Temperature (ºC)	Experimental mass loss (TGA, %)	Hypothesized process
0-170	9.8	Loss of methanol from structural pores (theoretical: 9.18% of mass)
170-400	44.8	Volatilization of 4,4'-bipy ligands (theoretical: 45.4% of mass)

D. Additional crystallographic information

a. *Hydrated and dehydrated crystals*

Table S4. Selected bond lengths at different temperatures.

Atom	Atom	Length (Å)
**Low temperature**		
Cu1	O8	2.241(6)
Cu1	N1	1.924(8)
Cu1	N4[1]	1.921(7)
Cu2	O13	2.356(8)
Cu2	N3	1.919(7)
Cu2	N2	1.896(7)
Cu3	O1[2]	2.309(9)
Cu3	N7	1.911(8)
Cu3	N6	1.897(8)
Cu4	N5	1.911(8)
Cu4	N8[1]	1.898(8)
**High temperature**		
Cu1	O2	2.460(3)
Cu1	O2[2]	2.460(3)
Cu1	N1[2]	1.9299(18)
Cu1	N1	1.9299(18)

[1]+X,1+Y,-1+Z, [2]1-X,+Y,3/2-Z

Table S5. Bond angles at different temperatures.

Atom	Atom	Atom	Angle (º)	Atom	Atom	Atom	Angle (º)
**Low temperature**							
N1	Cu1	O8	106.0(3)	C14	C15	C16	121.4(8)
N41	Cu1	O8	98.7(3)	C14	C15	C12	116.6(8)
N41	Cu1	N1	155.2(3)	N1	C10	C9	123.6(8)
N3	Cu2	O13	101.7(3)	C20	C19	C16	120.7(9)
N2	Cu2	O13	101.2(3)	C9	C6	C5	119.7(8)
N2	Cu2	N3	156.8(3)	C7	C6	C5	123.7(8)
N7	Cu3	O12	99.2(3)	C7	C6	C9	116.5(8)
N6	Cu3	O12	103.1(3)	N3	C11	C12	121.5(9)
N6	Cu3	N7	157.1(4)	C40	N8	Cu4[2]	124.5(8)
N8[1]	Cu4	N5	157.3(5)	C38	N8	Cu4[2]	119.6(8)
O8	P2	O7	107.5(4)	C38	N8	C40	115.8(9)
O8	P2	O5	109.8(4)	C2	C5	C6	118.2(7)
O6	P2	O8	114.2(4)	C4	C5	C6	125.4(8)
O6	P2	O7	109.6(4)	C4	C5	C2	116.3(8)
O6	P2	O5	109.2(5)	C29	C26	C25	120.9(9)

O7	P2	O5	106.2(5)	C29	C26	C27	118.7(9)
O15	P3	O14	105.4(5)	C27	C26	C25	120.3(10)
O16	P3	O15	112.2(5)	C10	C9	C6	119.7(9)
O16	P3	O13	114.3(5)	C19	C16	C15	121.8(8)
O16	P3	O14	109.4(4)	C19	C16	C17	116.4(8)
O13	P3	O15	109.1(4)	C17	C16	C15	121.8(8)
O13	P3	O14	106.0(6)	C34	C35	C36	127.0(10)
O12	P4	O11	108.9(4)	C34	C35	C32	115.0(9)
O12	P4	O9	114.5(5)	C32	C35	C36	118.0(9)
O12	P4	O10	108.1(6)	C24	C23	N6	124.3(10)
O9	P4	O11	111.7(5)	C37	C36	C35	127.5(10)
O9	P4	O10	109.2(4)	C37	C36	C39	115.3(9)
O10	P4	O11	103.8(5)	C39	C36	C35	117.1(9)
O2	P1	O4	107.0(5)	C35	C34	C33	124.6(11)
O2	P1	O3	110.5(4)	C26	C29	C30	119.5(9)
O4	P1	O3	109.7(6)	N2	C1	C2	124.1(8)
O1	P1	O2	112.2(6)	N8	C40	C39	125.9(11)
O1	P1	O4	109.4(4)	C23	C24	C25	120.4(10)
O1	P1	O3	108.1(5)	N4	C20	C19	123.4(8)
P2	O8	Cu1	121.8(4)	C24	C25	C26	121.5(10)
P3	O13	Cu2	115.8(5)	C22	C25	C26	123.0(10)
C10	N1	Cu1	124.1(6)	C22	C25	C24	115.5(9)
C10	N1	C8	117.3(8)	C15	C12	C11	120.7(9)
C8	N1	Cu1	118.5(7)	N3	C13	C14	123.8(9)
P4	O12	Cu3	115.4(5)	N5	C30	C29	121.8(10)
C11	N3	Cu2	121.1(6)	C1	C2	C5	118.2(8)
C13	N3	Cu2	121.5(6)	C6	C7	C8	120.1(9)
C13	N3	C11	117.2(8)	C31	C32	C35	120.2(11)
C33	N7	Cu3	120.1(7)	N2	C3	C4	121.9(9)
C33	N7	C31	117.7(9)	C18	C17	C16	117.9(10)
C31	N7	Cu3	122.0(7)	N8	C38	C37	124.3(11)
C1	N2	Cu2	119.3(6)	N7	C33	C34	120.4(10)
C1	N2	C3	116.3(8)	C36	C37	C38	120.9(11)
C3	N2	Cu2	124.2(6)	C13	C14	C15	120.1(9)
C20	N4	Cu1[2]	119.2(6)	C28	C27	C26	117.9(11)
C18	N4	Cu1[2]	125.5(7)	N5	C28	C27	124.9(11)
C18	N4	C20	115.3(8)	C40	C39	C36	117.6(10)
C30	N5	Cu4	121.2(8)	C32	C31	N7	122.1(11)
C28	N5	Cu4	121.6(7)	C5	C4	C3	123.0(9)
C28	N5	C30	117.1(9)	N1	C8	C7	122.6(9)
C23	N6	Cu3	122.3(7)	N6	C21	C22	123.5(13)
C21	N6	Cu3	122.8(8)	N4	C18	C17	126.2(10)
C21	N6	C23	114.7(9)	C25	C22	C21	121.6(12)
C12	C15	C16	122.1(9)				
**High temperature**							

O2	Cu1	O21	60.10(10)	O21	P1	O1A	109.7(3)
N1	Cu1	O2	102.79(8)	O2	P1	O1A[1]	109.7(3)
N1	Cu1	O21	102.02(8)	O21	P1	O1A[1]	104.8(10)
N11	Cu1	O2	102.02(8)	O2	P1	O1A	104.8(10)
N11	Cu1	O21	102.79(8)	O21	P1	O2	110.8(2)
N11	Cu1	N1	151.27(13)	P1	O2	Cu1	94.57(12)
O1A1	P1	O1A	117(2)	C1	N1	Cu1	122.39(18)
O1B	P1	O1A1	96(3)	C5	N1	Cu1	120.73(16)
O1B1	P1	O1A1	24.4(10)	C5	N1	C1	116.8(2)
O1B1	P1	O1A	96(3)	N1	C1	C2	123.0(2)
O1B	P1	O1A	24.4(10)	C1	C2	C3	120.3(2)
O1B1	P1	O1B	80(2)	C2	C3	C3[2]	121.7(3)
O1B1	P1	O21	127(2)	C2	C3	C4	116.3(2)
O1B	P1	O21	106.1(10)	C4	C3	C3[2]	121.9(2)
O1B	P1	O2	127(2)	C5	C4	C3	120.2(2)
O1B1	P1	O2	106.1(10)	N1	C5	C4	123.2(2)

Symmetry codes: low temperature: [1]+X,1+Y,-1+Z; [2]+X,-1+Y,1+Z; high temperature: [1]1-X,+Y,3/2-Z; [2]2-X,2-Y,1-Z

Table S6. Fractional Atomic Coordinates (×10^4) and Equivalent Isotropic Displacement Parameters (Å^2×10^3) for crystallographic data collected different temperatures.

Atom	*x*	*y*	*z*	U(eq)
**Low temperature**				
Cu1	-1255.1(16)	6489.3(8)	-129.4(8)	30.3(4)
Cu2	1301.9(18)	1468.0(9)	4838.9(8)	35.4(4)
Cu3	-1196(2)	3662.7(9)	5013.8(9)	41.5(4)
Cu4	1345(2)	8705.8(9)	46.0(9)	46.3(5)
P2	-4969(3)	6274.6(13)	-86.6(14)	28.4(5)
P3	5028(3)	1289.8(13)	4941.8(14)	29.9(5)
P4	-4839(3)	3725.9(13)	5096.3(16)	31.5(6)
P1	5048(3)	1262.5(14)	-15.4(16)	35.4(6)
O8	-3649(7)	6764(4)	123(4)	27.4(13)
O6	-5500(7)	5600(4)	570(4)	30.4(14)
O15	6285(9)	1880(4)	4563(5)	47(2)
O7	-4458(10)	5921(4)	-782(4)	47(2)
O16	5616(10)	613(4)	5565(4)	44.9(19)
O13	3748(8)	1771(4)	5201(5)	51(2)
N1	-691(9)	5554(4)	671(5)	30.0(17)
O12	-3625(10)	3328(4)	4742(5)	50(2)
O2	5959(10)	834(4)	-534(5)	53(2)
N3	794(9)	535(5)	5652(4)	27.5(16)
N7	-780(9)	2769(5)	5875(5)	30.9(17)
O14	4372(13)	943(4)	4270(5)	68(3)
O4	4102(11)	1918(5)	-556(5)	54(2)
N2	963(8)	2339(4)	3961(4)	25.7(16)

O11	-5829(10)	3057(5)	5628(5)	51(2)
N4	-915(8)	-2624(4)	8981(4)	26.0(16)
O9	-5790(10)	4332(4)	4511(5)	51(2)
O10	-4104(12)	4167(4)	5670(5)	58(2)
N5	908(12)	7810(5)	902(5)	43(2)
O5	-6319(9)	6854(5)	-391(6)	59(3)
O3	4004(10)	656(5)	528(5)	51(2)
N6	-773(10)	4586(5)	4198(5)	36.3(19)
O1	6053(11)	1655(4)	484(6)	63(3)
C15	101(11)	-742(5)	6941(5)	26.1(18)
C10	723(10)	5363(5)	888(5)	27.0(19)
C19	868(10)	-1836(6)	8089(5)	29.1(19)
C6	-48(10)	4245(5)	1926(5)	26.2(18)
C11	-650(11)	365(6)	5872(6)	34(2)
N8	937(11)	-390(5)	9205(5)	41(2)
C5	358(9)	3594(5)	2625(5)	22.2(17)
C26	248(12)	6541(5)	2197(5)	31(2)
C9	1105(10)	4737(6)	1496(5)	29.0(19)
C16	-250(11)	-1397(5)	7639(5)	28.1(19)
C35	-95(12)	1541(5)	7198(6)	32(2)
C23	667(12)	4826(6)	3982(6)	37(2)
C36	161(12)	875(5)	7891(5)	31(2)
C34	956(13)	1991(6)	6746(6)	38(2)
C29	1738(12)	6743(6)	1985(6)	35(2)
C1	-471(11)	2561(6)	3747(6)	35(2)
C40	1970(13)	66(7)	8790(7)	45(3)
C24	1025(12)	5432(6)	3362(6)	36(2)
C20	515(10)	-2432(6)	8740(6)	31(2)
C25	-117(13)	5871(6)	2892(6)	38(2)
C12	-1019(12)	-271(6)	6504(6)	37(2)
C13	1872(11)	65(6)	6067(6)	37(2)
C30	2038(12)	7368(6)	1331(6)	38(2)
C2	-848(10)	3169(6)	3103(6)	34(2)
C7	-1494(11)	4416(7)	1675(7)	43(3)
C32	-1613(15)	1732(6)	6957(6)	44(3)
C3	2068(12)	2726(7)	3480(7)	47(3)
C17	-1742(13)	-1585(7)	7888(6)	47(3)
C38	-444(15)	-212(6)	8998(6)	45(3)
C33	657(12)	2595(6)	6107(7)	39(2)
C37	-873(14)	425(6)	8347(6)	43(3)
C14	1572(11)	-566(6)	6696(6)	38(2)
C27	-912(15)	6995(6)	1756(6)	45(3)
C28	-517(14)	7602(6)	1125(6)	42(3)
C39	1721(13)	690(7)	8129(7)	48(3)
C31	-1905(14)	2328(6)	6311(6)	44(3)

C4	1761(11)	3352(7)	2843(7)	50(3)
C8	-1790(11)	5080(7)	1062(7)	44(3)
C21	-1834(17)	5012(8)	3755(8)	63(4)
C18	-1962(13)	-2192(7)	8560(7)	49(3)
C22	-1535(16)	5653(7)	3116(8)	59(3)
**High temperature**				
Cu1	5000	8388.2(6)	7500	69.1(3)
P1	5000	4974.9(10)	7500	69.3(3)
O1A	3700(40)	4010(20)	8167(9)	87(4)
O1B	4440(60)	3690(20)	8290(30)	69(7)
O2	3989(3)	5949(3)	6164(2)	90.3(7)
N1	6690(2)	8937(2)	6509(2)	55.6(5)
C1	7609(3)	7888(3)	6045(4)	72.6(7)
C2	8892(4)	8263(3)	5442(4)	69.8(7)
C3	9295(2)	9777(2)	5303(2)	49.2(5)
C4	8319(3)	10857(3)	5753(3)	57.0(5)
C5	7052(3)	10401(3)	6342(3)	57.4(5)

Ueq is defined as 1/3 of the trace of the orthogonalised U_{IJ} tensor

Table S7. Anisotropic Displacement Parameters ($Å^2×10^3$) for crystallographic data at different temperatures.

Atom	U_{11}	U_{22}	U_{33}	U_{23}	U_{13}	U_{12}
**Low temperature**						
Cu1	37.6(8)	20.7(7)	27.1(7)	5.7(5)	-0.2(6)	-0.9(5)
Cu2	42.7(9)	28.0(7)	27.4(7)	9.2(6)	3.2(6)	-0.3(6)
Cu3	58.1(11)	30.3(8)	27.9(8)	9.3(6)	-2.4(7)	-3.8(7)
Cu4	63.8(11)	27.7(8)	37.5(9)	12.2(7)	-0.4(8)	5.1(7)
P2	34.9(13)	17.2(10)	32.7(12)	-5.3(9)	-6.7(10)	-1.0(9)
P3	38.8(14)	17.6(10)	31.4(12)	-2.2(9)	2.1(10)	-7.2(9)
P4	32.6(13)	16.7(10)	43.9(14)	-4.8(10)	2.8(10)	-1.5(9)
P1	47.5(15)	16.8(10)	40.3(14)	-3.6(10)	-5.6(11)	5.4(10)
O8	25(3)	24(3)	35(3)	-10(3)	-1(3)	-4(2)
O6	27(3)	27(3)	37(4)	-9(3)	14(3)	-6(3)
O15	47(5)	30(4)	69(5)	-19(4)	28(4)	-8(3)
O7	84(6)	27(4)	27(4)	-2(3)	6(4)	0(4)
O16	81(6)	24(3)	32(4)	-11(3)	-8(4)	8(3)
O13	31(4)	28(4)	85(6)	2(4)	9(4)	0(3)
N1	33(4)	20(4)	35(4)	-2(3)	5(3)	5(3)
O12	64(5)	19(3)	66(5)	-11(3)	30(4)	-3(3)
O2	56(5)	28(4)	70(6)	-3(4)	24(4)	12(3)
N3	30(4)	27(4)	18(3)	10(3)	2(3)	-4(3)
N7	33(4)	26(4)	30(4)	2(3)	8(3)	-2(3)
O14	118(8)	17(3)	64(6)	1(4)	-52(6)	-3(4)
O4	79(6)	31(4)	56(5)	-21(4)	-27(5)	25(4)

N2	14(3)	29(4)	30(4)	-1(3)	2(3)	0(3)
O11	56(5)	32(4)	61(5)	-5(4)	23(4)	-7(4)
N4	27(4)	15(3)	30(4)	6(3)	2(3)	5(3)
O9	68(6)	26(4)	55(5)	-5(3)	-22(4)	12(4)
O10	88(7)	23(4)	60(5)	-3(4)	-27(5)	4(4)
N5	82(7)	20(4)	25(4)	-1(3)	-13(4)	-2(4)
O5	36(4)	35(4)	112(8)	-30(5)	-46(5)	16(3)
O3	56(5)	42(4)	67(5)	-38(4)	14(4)	-11(4)
N6	48(5)	25(4)	35(4)	-6(3)	-2(4)	4(4)
O1	81(7)	25(4)	80(6)	-4(4)	-48(5)	7(4)
C15	33(5)	17(4)	25(4)	1(3)	-5(4)	0(3)
C10	23(4)	27(4)	27(4)	1(4)	6(3)	5(3)
C19	23(4)	32(5)	30(5)	-3(4)	-6(4)	-4(4)
C6	18(4)	27(4)	30(5)	-1(4)	1(3)	5(3)
C11	33(5)	25(5)	38(5)	5(4)	-3(4)	2(4)
N8	61(6)	21(4)	32(4)	10(3)	-13(4)	-4(4)
C5	11(4)	23(4)	27(4)	4(3)	6(3)	2(3)
C26	50(6)	16(4)	26(5)	-2(3)	1(4)	7(4)
C9	20(4)	32(5)	33(5)	-5(4)	5(4)	-3(4)
C16	31(5)	18(4)	31(5)	4(4)	6(4)	-3(3)
C35	40(6)	23(4)	32(5)	-7(4)	-1(4)	5(4)
C23	43(6)	35(5)	29(5)	-2(4)	-5(4)	-1(4)
C36	43(6)	24(4)	26(5)	-7(4)	3(4)	-9(4)
C34	46(6)	39(6)	26(5)	-1(4)	-4(4)	-9(5)
C29	33(5)	31(5)	37(5)	3(4)	-13(4)	6(4)
C1	24(5)	39(5)	32(5)	10(4)	2(4)	-6(4)
C40	37(6)	38(6)	47(6)	12(5)	1(5)	8(5)
C24	42(6)	37(5)	27(5)	0(4)	-5(4)	-9(4)
C20	20(4)	31(5)	38(5)	2(4)	-12(4)	-3(4)
C25	59(7)	21(4)	31(5)	-1(4)	5(5)	-8(4)
C12	33(5)	36(5)	33(5)	8(4)	6(4)	3(4)
C13	19(4)	45(6)	38(5)	7(4)	3(4)	9(4)
C30	38(6)	31(5)	38(5)	4(4)	8(4)	4(4)
C2	11(4)	35(5)	44(6)	14(4)	11(4)	9(4)
C7	15(4)	45(6)	54(7)	18(5)	-3(4)	-6(4)
C32	74(8)	23(5)	30(5)	7(4)	7(5)	-5(5)
C3	23(5)	49(6)	50(7)	24(5)	0(4)	-9(4)
C17	48(6)	36(6)	38(6)	27(5)	6(5)	11(5)
C38	74(9)	33(5)	25(5)	-3(4)	8(5)	-4(5)
C33	33(5)	32(5)	48(6)	-5(5)	6(4)	3(4)
C37	54(7)	32(5)	35(6)	8(4)	-1(5)	6(5)
C14	21(5)	37(5)	47(6)	8(5)	5(4)	5(4)
C27	68(8)	22(5)	37(6)	6(4)	10(5)	-2(5)
C28	59(7)	31(5)	28(5)	8(4)	3(5)	-3(5)
C39	41(6)	45(6)	47(7)	9(5)	1(5)	9(5)

C31	52(7)	34(5)	40(6)	4(5)	6(5)	-1(5)
C4	17(5)	55(7)	56(7)	27(6)	-5(4)	-4(4)
C8	18(5)	42(6)	58(7)	14(5)	1(4)	-6(4)
C21	70(7)	47(6)	59(6)	14(5)	6(6)	-18(5)
C18	34(5)	48(6)	48(6)	23(5)	1(4)	-4(4)
C22	60(6)	43(6)	56(6)	24(5)	-6(5)	0(5)
**High temperature**						
Cu1	66.5(4)	58.2(4)	101.2(5)	0	54.6(3)	0
P1	122.6(8)	42.2(4)	56.8(5)	0	47.5(5)	0
O1A	117(10)	87(4)	60(2)	2(2)	30(3)	-40(6)
O1B	98(15)	55(6)	62(5)	-9(3)	36(7)	-28(6)
O2	136.3(19)	78.5(13)	75.6(12)	16.3(10)	62.3(12)	37.8(13)
N1	54.6(10)	52.3(11)	69.0(12)	-0.8(8)	32.0(9)	-2.5(8)
C1	80.5(17)	49.1(12)	109(2)	-4.7(13)	60.2(16)	-7.3(12)
C2	79.2(17)	48.4(13)	103(2)	-6.9(12)	60.4(16)	-1.9(11)
C3	50.2(11)	51.1(11)	51.9(10)	0.9(9)	23.5(9)	-2.2(9)
C4	55.6(12)	46.5(11)	76.7(14)	6.1(10)	31.4(11)	1.5(9)
C5	53.4(11)	52.0(12)	74.2(14)	3.1(10)	30.1(10)	6.5(10)

The Anisotropic displacement factor exponent takes the form: $-2\pi^2[h^2a^{*2}U_{11}+2hka^*b^*U_{12}+\dots]$.

Table S8. Bond lengths for crystallographic data at different temperatures.

Atom	Atom	Length (Å)	Atom	Atom	Length (Å)
**Low temperature**					
Cu1	O8	2.241(6)	N6	C21	1.326(16)
Cu1	N1	1.925(8)	C15	C16	1.483(11)
Cu1	N4[1]	1.922(7)	C15	C12	1.390(13)
Cu2	O13	2.356(8)	C15	C14	1.385(13)
Cu2	N3	1.919(7)	C10	C9	1.362(13)
Cu2	N2	1.895(7)	C19	C16	1.377(13)
Cu3	O12	2.310(9)	C19	C20	1.374(13)
Cu3	N7	1.912(8)	C6	C5	1.494(12)
Cu3	N6	1.896(8)	C6	C9	1.419(12)
Cu4	N5	1.910(8)	C6	C7	1.367(13)
Cu4	N8[1]	1.899(8)	C11	C12	1.394(13)
P2	O8	1.531(6)	N8	C40	1.305(14)
P2	O6	1.498(6)	N8	C38	1.295(16)
P2	O7	1.559(8)	C5	C2	1.444(11)
P2	O5	1.562(7)	C5	C4	1.339(12)
P3	O15	1.540(7)	C26	C29	1.393(15)
P3	O16	1.485(7)	C26	C25	1.497(13)
P3	O13	1.531(8)	C26	C27	1.406(15)
P3	O14	1.560(8)	C16	C17	1.407(14)
P4	O12	1.486(8)	C35	C36	1.474(13)
P4	O11	1.559(8)	C35	C34	1.340(14)

P4	O9	1.529(8)	C35	C32	1.427(16)
P4	O10	1.543(9)	C23	C24	1.348(14)
P1	O2	1.530(8)	C36	C37	1.333(14)
P1	O4	1.533(8)	C36	C39	1.460(15)
P1	O3	1.538(9)	C34	C33	1.358(14)
P1	O1	1.519(8)	C29	C30	1.394(13)
N1	C10	1.330(12)	C1	C2	1.384(13)
N1	C8	1.345(12)	C40	C39	1.395(14)
N3	C11	1.349(12)	C24	C25	1.407(15)
N3	C13	1.347(12)	C25	C22	1.343(17)
N7	C33	1.351(13)	C13	C14	1.371(14)
N7	C31	1.369(13)	C7	C8	1.389(14)
N2	C1	1.356(12)	C32	C31	1.358(14)
N2	C3	1.359(12)	C3	C4	1.380(14)
N4	C20	1.352(12)	C17	C18	1.388(13)
N4	C18	1.303(13)	C38	C37	1.429(14)
N5	C30	1.370(14)	C27	C28	1.371(13)
N5	C28	1.346(15)	C21	C22	1.393(15)
N6	C23	1.367(14)			
**High temperature**					
Cu1	O2	2.460(3)	P1	O2[1]	1.497(2)
Cu1	O2[1]	2.460(3)	N1	C1	1.338(3)
Cu1	N1[1]	1.9299(18)	N1	C5	1.332(3)
Cu1	N1	1.9299(18)	C1	C2	1.377(4)
P1	O1A[1]	1.624(17)	C2	C3	1.380(3)
P1	O1A	1.624(17)	C3	C3[2]	1.493(4)
P1	O1B[1]	1.457(13)	C3	C4	1.384(3)
P1	O1B	1.457(13)	C4	C5	1.376(3)
P1	O2	1.497(2)			

Symmetry codes: [1]+X,1+Y,-1+Z, [2]1-X,+Y,3/2-Z

Table S9. Torsion angles for crystallographic data at different temperatures.

A	B	C	D	Angle (˚)	A	B	C	D	Angle (˚)
**Low temperature**									
Cu1	N1	C10	C9	174.6(7)	C16	C15	C12	C11	177.0(10)
Cu1	N1	C8	C7	-176.7(10)	C16	C15	C14	C13	-177.3(10)
Cu1[1]	N4	C20	C19	176.7(8)	C16	C19	C20	N4	-0.2(16)
Cu1[1]	N4	C18	C17	-175.5(11)	C16	C17	C18	N4	-2(2)
Cu2	N3	C11	C12	-174.6(8)	C35	C36	C37	C38	179.2(10)
Cu2	N3	C13	C14	174.3(9)	C35	C36	C39	C40	179.2(10)
Cu2	N2	C1	C2	-177.8(9)	C35	C34	C33	N7	-1.6(17)
Cu2	N2	C3	C4	179.3(10)	C35	C32	C31	N7	-0.6(17)
Cu3	N7	C33	C34	176.2(8)	C23	N6	C21	C22	0(2)
Cu3	N7	C31	C32	-175.0(9)	C23	C24	C25	C26	-179.4(10)

Cu3	N6	C23	C24	173.7(8)	C23	C24	C25	C22	1.2(16)
Cu3	N6	C21	C22	-174.8(11)	C36	C35	C34	C33	179.3(10)
Cu4	N5	C30	C29	176.2(8)	C36	C35	C32	C31	-178.4(10)
Cu4	N5	C28	C27	-176.5(9)	C34	C35	C36	C37	179.8(11)
Cu4[1]	N8	C40	C39	174.2(10)	C34	C35	C36	C39	1.7(15)
Cu4[1]	N8	C38	C37	-176.2(9)	C34	C35	C32	C31	0.2(15)
O6	P2	O8	Cu1	95.8(5)	C29	C26	C25	C24	0.9(15)
O15	P3	O13	Cu2	131.8(5)	C29	C26	C25	C22	-179.7(12)
O7	P2	O8	Cu1	-26.1(5)	C29	C26	C27	C28	1.7(15)
O16	P3	O13	Cu2	-101.8(5)	C1	N2	C3	C4	4.9(18)
O13	Cu2	N2	C1	-156.3(8)	C40	N8	C38	C37	1.6(17)
O13	Cu2	N2	C3	29.4(10)	C24	C25	C22	C21	-2(2)
N1	C10	C9	C6	0.7(15)	C20	N4	C18	C17	2.3(19)
O12	Cu3	N6	C23	-158.2(8)	C20	C19	C16	C15	179.0(9)
O12	Cu3	N6	C21	16.4(11)	C20	C19	C16	C17	0.8(15)
N3	Cu2	N2	C1	13.7(14)	C25	C26	C29	C30	179.7(9)
N3	Cu2	N2	C3	-160.6(10)	C25	C26	C27	C28	179.9(10)
N3	C11	C12	C15	1.4(17)	C12	C15	C16	C19	-177.0(10)
N3	C13	C14	C15	-0.8(18)	C12	C15	C16	C17	1.1(15)
N7	Cu3	N6	C23	8.4(15)	C12	C15	C14	C13	2.2(16)
N7	Cu3	N6	C21	-177.0(11)	C13	N3	C11	C12	0.1(15)
O14	P3	O13	Cu2	18.9(6)	C30	N5	C28	C27	1.4(17)
N2	C1	C2	C5	0.2(17)	C2	C5	C4	C3	0.9(19)
N2	C3	C4	C5	-4(2)	C7	C6	C5	C2	-5.7(15)
O11	P4	O12	Cu3	-121.0(5)	C7	C6	C5	C4	172.7(12)
O9	P4	O12	Cu3	113.0(5)	C7	C6	C9	C10	2.7(15)
O10	P4	O12	Cu3	-8.8(6)	C32	C35	C36	C37	-1.8(16)
O5	P2	O8	Cu1	-141.3(5)	C32	C35	C36	C39	-179.9(10)
N6	C23	C24	C25	0.7(17)	C32	C35	C34	C33	0.8(16)
N6	C21	C22	C25	2(2)	C3	N2	C1	C2	-3.0(16)
C15	C16	C17	C18	-178.2(11)	C38	N8	C40	C39	-3.6(19)
C10	N1	C8	C7	0.3(17)	C33	N7	C31	C32	-0.1(16)
C19	C16	C17	C18	0.0(17)	C37	C36	C39	C40	0.9(16)
C6	C5	C2	C1	179.4(9)	C14	C15	C16	C19	2.5(15)
C6	C5	C4	C3	-177.5(12)	C14	C15	C16	C17	-179.4(11)
C6	C7	C8	N1	3(2)	C14	C15	C12	C11	-2.5(15)
C11	N3	C13	C14	-0.4(16)	C27	C26	C29	C30	-2.1(15)
N8	C40	C39	C36	2(2)	C27	C26	C25	C24	-177.2(10)
N8	C38	C37	C36	1.6(18)	C27	C26	C25	C22	2.2(16)
C5	C6	C9	C10	-175.8(9)	C28	N5	C30	C29	-1.7(15)
C5	C6	C7	C8	174.0(11)	C39	C36	C37	C38	-2.6(16)
C26	C29	C30	N5	2.2(16)	C31	N7	C33	C34	1.1(15)
C26	C25	C22	C21	178.3(12)	C4	C5	C2	C1	0.9(16)
C26	C27	C28	N5	-1.4(17)	C8	N1	C10	C9	-2.2(15)
C9	C6	C5	C2	172.7(9)	C21	N6	C23	C24	-1.3(16)

C9	C6	C5	C4	-8.9(16)	C18	N4	C20	C19	-1.3(15)
C9	C6	C7	C8	-4.4(17)					
**High temperature**									
Cu1	N1	C1	C2	-175.3(2)	C1	N1	C5	C4	-1.2(4)
Cu1	N1	C5	C4	175.21(18)	C1	C2	C3	C3^{3}	178.3(3)
O1A	P1	O2	Cu1	118.2(7)	C1	C2	C3	C4	-1.8(4)
O1A^{2}	P1	O2	Cu1	-115.2(13)	C2	C3	C4	C5	1.7(4)
O1B	P1	O2	Cu1	130.6(11)	C3^{3}	C3	C4	C5	-178.5(2)
O1B^{2}	P1	O2	Cu1	-141(2)	C3	C4	C5	N1	-0.2(4)
O2^{3}	P1	O2	Cu1	0.000(1)	C5	N1	C1	C2	1.0(4)
N1	C1	C2	C3	0.5(5)					

Symmetry operations: ^{1}+X,-1+Y,1+Z, ^{2}1-X,+Y,3/2-Z; ^{3}2-X,2-Y,1-Z

Table S10. Hydrogen Atom Coordinates (Å×10^{4}) and Isotropic Displacement Parameters (Å^{2}×10^{3}) for crystallographic data at different temperatures

Atom	*x*	*y*	*z*	U(eq)
**Low temperature**				
H15	5994.84	2360.40	4520.33	71
H14	4631.40	455.70	4333.72	102
H4A	3520.27	2133.88	-288.67	80
H11A	-5887.96	2653.38	5431.37	77
H10A	-4152.43	4671.57	5486.65	88
H10	1510.92	5682.28	603.98	32
H19	1894.70	-1726.38	7948.69	35
H11	-1434.53	686.23	5589.29	41
H9	2132.97	4630.40	1630.72	35
H23	1469.64	4547.77	4287.70	44
H34	1982.46	1879.12	6881.42	46
H29	2542.28	6458.30	2283.26	43
H1	-1267.90	2281.55	4059.47	42
H40	2985.90	-36.44	8952.41	53
H24	2052.72	5562.26	3241.89	44
H20	1316.10	-2723.92	9035.31	38
H12	-2047.43	-384.05	6637.13	44
H13	2893.55	175.47	5917.44	44
H30	3055.88	7490.54	1179.69	45
H2	-1871.71	3303.19	2979.00	41
H7	-2298.51	4079.98	1919.87	52
H32	-2421.79	1440.76	7249.75	53
H3	3084.21	2559.42	3585.91	56
H17	-2570.96	-1306.85	7605.45	56
H38	-1222.84	-523.53	9297.35	54
H33	1462.82	2896.29	5822.56	46
H37	-1911.42	528.89	8238.25	52

H14A	2376.45	-882.45	6963.35	46
H27	-1940.56	6883.32	1891.32	54
H28	-1307.59	7897.89	823.22	50
H39	2535.96	983.36	7844.20	58
H31	-2922.60	2441.61	6157.50	53
H4	2581.93	3624.30	2545.83	60
H8	-2807.97	5203.31	914.13	53
H21	-2857.06	4872.99	3879.37	76
H18	-2973.13	-2302.33	8730.18	59
H22	-2352.13	5941.37	2832.08	70
**High temperature**				
H1A	3930(60)	3920(50)	9110(60)	114(14)
H1	7370.52	6857.24	6133.35	87
H2	9489.53	7491.36	5127.12	84
H4	8519.19	11895.25	5656.14	68
H5	6418.22	11151.29	6637.87	69

Table S11. Solvent mask information for crystal refinement in hydrated structure

Number	X	Y	Z	Volume	Electron count
1	0.483	-0.208	0.750	204.0	51.0
2	0.517	-0.393	0.250	204.0	50.9

b. ***MeOH-absorbed crystals***

Table S13. Bond angles of MeOH-absorbed crystal.

Atom	Atom	Atom	Angle (º)	Atom	Atom	Atom	Angle (º)
N1	Cu1	O1	101.3(3)	C3	C5	C6	122.2(7)
N21	Cu1	N1	156.1(3)	C10	C8	C6	120.2(8)
N21	Cu1	O1	102.5(3)	C8	C6	C5	121.3(7)
C1	N1	Cu1	123.6(6)	C7	C6	C5	122.7(7)
C2	N1	Cu1	119.5(6)	C7	C6	C8	116.1(8)
C2	N1	C1	116.6(7)	C2	C4	C5	119.5(8)
O2	P14	O4	104.9(4)	N1	C1	C3	122.7(8)
O3	P14	O2	110.4(4)	N2	C10	C8	123.8(8)
O3	P14	O4	109.4(3)	P14	O1	Cu1	122.8(3)
O3	P14	O1	114.5(4)	N1	C2	C4	124.2(8)
O1	P14	O2	108.7(4)	C1	C3	C5	120.5(8)
O1	P14	O4	108.5(3)	C9	N2	$Cu1^2$	122.3(6)
N2	C9	C7	122.4(8)	C10	N2	$Cu1^2$	120.8(6)
C4	C5	C6	121.3(7)	C10	N2	C9	116.9(7)
C4	C5	C3	116.5(8)	C6	C7	C9	120.5(8)

Table S14. Fractional Atomic Coordinates (×10^4) and Equivalent Isotropic Displacement Parameters (Å^2×10^3) for crystallographic data of MeOH-absorbed crystal.

Atom	*x*	*y*	*z*	U(eq)
Cu1	3214.4(11)	1243.1(11)	8166.7(13)	30.7(4)
N1	4876(7)	716(8)	7167(9)	28.8(15)
P14	2946(2)	4867(2)	7228(3)	27.2(4)
C9	10700(10)	-2007(9)	4594(12)	32.0(18)
C5	7500(8)	53(10)	6002(10)	26.8(15)
C8	9801(10)	877(10)	4962(13)	31.9(18)
C6	8895(9)	-285(8)	5393(11)	27.9(16)
C4	7089(12)	1528(11)	6267(15)	41(2)
C1	5261(10)	-723(10)	6898(13)	33.4(19)
O2	2290(10)	6227(6)	8033(9)	44.8(17)
O5	8673(10)	4648(10)	5589(11)	63(2)
O4	4320(7)	5543(7)	6581(8)	37.1(14)
O3	1748(6)	4267(7)	5825(8)	35.1(13)
C10	11063(10)	532(9)	4346(12)	33(2)
O1	3589(7)	3707(6)	8540(8)	29.5(13)
C2	5802(11)	1793(10)	6845(13)	40(2)
C3	6529(10)	-1079(9)	6336(13)	33(2)
C11	8020(20)	4660(20)	3970(20)	96(5)
N2	11511(8)	-878(8)	4119(10)	31.0(16)

C7	9402(10)	-1743(10)	5238(11)	32.5(18)

Table S15. Anisotropic Displacement Parameters ($Å^2×10^3$) for crystallographic data of MeOH-absorbed crystal.

Atom	U_{11}	U_{22}	U_{33}	U_{23}	U_{13}	U_{12}
Cu1	24.6(5)	37.8(6)	34.9(7)	-2.4(5)	17.2(4)	-0.8(5)
N1	21(3)	36(3)	32(4)	-10(3)	12(3)	0(3)
P14	30.3(9)	30.9(9)	23.7(9)	0.2(8)	13.2(7)	0.2(7)
C9	30(4)	29(4)	42(5)	4(3)	18(4)	1(3)
C5	21(3)	35(4)	28(4)	2(3)	12(3)	-5(3)
C8	29(4)	32(4)	42(5)	4(4)	21(4)	-1(3)
C6	28(4)	28(3)	32(4)	-3(3)	14(3)	0(3)
C4	38(5)	32(4)	61(7)	-6(4)	27(5)	-1(3)
C1	32(4)	30(4)	42(5)	-1(3)	18(4)	-1(3)
O2	78(5)	35(3)	28(4)	5(2)	26(3)	20(3)
O5	58(3)	60(3)	71(3)	1(2)	14(2)	-2(2)
O4	31(3)	52(3)	29(3)	0(3)	8(3)	-14(3)
O3	27(3)	51(3)	30(3)	-7(3)	12(2)	-8(2)
C10	31(4)	32(4)	42(5)	2(3)	18(4)	0(3)
O1	37(3)	35(3)	22(3)	0(2)	17(2)	5(2)
C2	36(4)	34(4)	56(6)	-11(4)	24(4)	0(3)
C3	28(4)	29(4)	46(6)	-3(4)	15(4)	4(3)
C11	97(5)	93(5)	97(6)	1(2)	20(3)	-1(2)
N2	27(3)	29(3)	41(5)	2(3)	15(3)	0(3)
C7	33(4)	32(4)	39(5)	3(4)	21(4)	-2(3)

Table S16. Bond lengths for crystallographic data of MeOH-absorbed crystal.

Atom	Atom	Length (Å)	Atom	Atom	Length (Å)
Cu1	N1	1.929(7)	C5	C6	1.486(9)
Cu1	O1	2.228(5)	C5	C4	1.394(11)
Cu1	N2[1]	1.908(8)	C5	C3	1.400(12)
N1	C1	1.358(11)	C8	C6	1.412(11)
N1	C2	1.335(12)	C8	C10	1.382(13)
P14	O2	1.567(6)	C6	C7	1.390(11)
P14	O4	1.575(6)	C4	C2	1.370(14)
P14	O3	1.502(6)	C1	C3	1.363(14)
P14	O1	1.528(6)	O5	C11	1.356(19)
C9	N2	1.352(11)	C10	N2	1.344(11)
C9	C7	1.411(12)			

Symmetry operations: [1]-1+X,-Y,1/2+Z

Table S17. Torsion angles for crystallographic data of MeOH-absorbed crystal.

A	B	C	D	Angle (˚)	A	B	C	D	Angle (˚)
Cu1	N1	C1	C3	-173.4(8)	C1	N1	C2	C4	0.2(16)
Cu1	N1	C2	C4	174.2(9)	O2	P14	O1	Cu1	137.6(4)
N1	C1	C3	C5	-0.2(16)	O4	P14	O1	Cu1	-108.9(4)
C5	C6	C7	C9	177.5(8)	O3	P14	O1	Cu1	13.6(5)
C5	C4	C2	N1	-0.8(17)	C10	C8	C6	C5	-177.8(8)
C8	C6	C7	C9	-3.6(12)	C10	C8	C6	C7	3.3(13)
C8	C10	N2	Cu1[1]	177.1(7)	C2	N1	C1	C3	0.4(15)
C8	C10	N2	C9	-2.8(14)	C3	C5	C6	C8	175.7(10)
C6	C5	C4	C2	-179.3(9)	C3	C5	C6	C7	-5.4(13)
C6	C5	C3	C1	179.8(8)	C3	C5	C4	C2	0.9(15)
C6	C8	C10	N2	-0.1(15)	N2	C9	C7	C6	0.7(14)
C4	C5	C6	C8	-4.0(13)	C7	C9	N2	Cu1[1]	-177.4(7)
C4	C5	C6	C7	174.8(10)	C7	C9	N2	C10	2.5(14)
C4	C5	C3	C1	-0.4(14)					

[1]1+X,-Y,-1/2+Z

Table S18. Hydrogen Atom Coordinates (Å×10^4) and Isotropic Displacement Parameters (Å^2×10^3) for crystallographic data of MeOH-absorbed crystal.

Atom	*x*	*y*	*z*	U(eq)
H9	11015.93	-3015.2	4489.55	38
H8	9543.52	1897.56	5096.65	38
H4	7696.84	2343.48	6047.07	49
H1	4620.6	-1514.59	7109.38	40
H2	2258.15	6001.46	8987.86	67
H5	9629.26	4543.72	5732.25	95
H4A	3991.58	5855.06	5627.15	56
H10	11647.47	1339.9	4065.54	40
H2A	5554.37	2806.78	7028.23	48
H3	6754.13	-2103	6169.27	40
H11A	8063.92	3650.77	3528.04	144
H11B	8581.29	5364.41	3428.89	144
H11C	6946.2	4983.26	3799.56	144
H7	8871	-2568.09	5568.08	39

E. References

1. Sheldrick, G. M. SHELXL-97, A program for crystal structure refinement Göttingen, **1997**.
2. Sheldrick, G. M. Crystal structure refinement with SHELXL. *Acta. Cryst*. **2015**, C71, 3-8.
3. Sheldrick, G. M. SHELXT- Integrated space-group and crystal-structure determination. *Acta. Cryst*. **2015**, A71, 3-8.
4. Dolomanov, O. V., Bourhis, L. J., Gildea, R. J., Howard, J. A. K., Puschmann, H. OLEX2: a complete structure solution, refinement and analysis program. *J. Appl. Cryst*. **2009**, 42, 339-341.
